# Re-development of mental health first aid guidelines for suicidal ideation and behaviour: a Delphi study

**DOI:** 10.1186/s12888-014-0241-8

**Published:** 2014-09-13

**Authors:** Anna M Ross, Claire M Kelly, Anthony F Jorm

**Affiliations:** Melbourne School of Population and Global Health, Level 4, 207 Bouverie St, The University of Melbourne, Melbourne, Victoria 3010 Australia; Mental Health First Aid Australia, Level 6, 369 Royal Parade, Parkville, Victoria 3052 Australia; School of Psychology, Deakin University, Burwood, Victoria 3125 Australia

**Keywords:** Suicide, Mental health first aid, Prevention, Helping behaviour, Assistance

## Abstract

**Background:**

Suicide continues to be a leading cause of death globally. Friends and family are considered best positioned to provide initial assistance if someone is suicidal. Expert consensus guidelines on how to do this were published in 2008. Re-developing these guidelines is necessary to ensure they contain the most current recommended helping actions and remain consistent with the suicide prevention literature.

**Methods:**

The Delphi consensus method was used to determine the importance of including helping statements in the guidelines. These statements describe helping actions a member of the public can take, and information they should have, to help someone who is experiencing suicidal thoughts. Systematic searches of the available suicide prevention literature were carried out to find helping statements. Two expert panels, comprising 41 suicide prevention professionals and 35 consumer advocates respectively, rated each statement. Statements were accepted for inclusion in the guidelines if they were endorsed by at least 80% of each panel.

**Results:**

Out of 436 statements, 164 were endorsed as appropriate helping actions in providing assistance to someone experiencing suicidal thoughts or engaging in suicidal behaviour. These statements were used to form the re-developed guidelines.

**Conclusion:**

The re-development of the guidelines has resulted in more comprehensive guidance than the earlier version, with the endorsement of 164 helping actions, compared to 30 previously. These guidelines will form the basis of a suicide prevention course aimed at educating members of the public on providing first aid to someone who is experiencing suicidal thoughts.

**Electronic supplementary material:**

The online version of this article (doi:10.1186/s12888-014-0241-8) contains supplementary material, which is available to authorized users.

## Background

Suicide remains a mental health problem of particular concern, being a leading cause of death worldwide [1]. Rates of death by suicide have been estimated to be as high as 10.5 to 11.5 per 100,000 in western countries [2,3]. The number of suicide attempts made is significantly higher, estimated to be 25 more attempts made per death [4]. While the number of completed suicides is not large in comparison to the prevalence of other mental health problems, the full impact of these deaths is through their devastating effects on friends, relatives and the wider community [5].

As proposed by the interpersonal theory [6], suicide is considered to be influenced by three different components; behavioural, emotional, and social. The theory proposes that feelings of being a burden to loved ones (emotional), and feelings of social disconnection and lack of belongingness (social), result in suicidal desire. The ability to enact lethal self-injury (behavioural) based on this desire ultimately determines whether suicide is carried out. Social isolation has been revealed to be one of the strongest and most reliable predictors of suicide [6]. Intervention through social factors is therefore an avenue for suicide prevention, by which others can act to reduce suicide risk. By increasing feelings of belongingness by connecting with the person, and by furthering this connectedness through linking them with additional social and professional support, others can play a central role in reducing suicide risk.

Close friends and family members are well placed to take on this role [7]. As well as being prime sources of emotional support, they are often in the best position to notice and act on warning signs of suicide in a loved one. However, it is common for members of the public to be unsure of what the warning signs for suicide are, or what to do if they are worried that someone might be suicidal [8,9]. It is therefore important that guidelines that contain the most current and relevant helping recommendations are available to provide guidance to the general public in assisting someone who is suicidal.

Mental health first aid guidelines have been developed through a series of Delphi expert consensus studies to provide recommendations to members of the public on providing assistance to a person with a mental health problem, including depression, psychosis, substance use or eating disorders, or experiencing a mental health crisis, such as having suicidal ideation, experiencing a traumatic event or a panic attack, or engaging in non-suicidal self-injury [10–17]. These guidelines were used to inform the content of the 2^nd^ edition Mental Health First Aid (MHFA) course [18]. The programme teaches adult members of the public how to provide assistance to someone who has a mental health problem or is experiencing a mental health crisis, until appropriate professional assistance is received or the crisis resolves [19]. While suicide prevention is only briefly covered, this course has been found to be effective in providing the knowledge required to intervene and increasing helping behaviours [20].

The guidelines for assisting a person with suicidal thoughts and behaviours were developed in 2008. As well as informing the content of MHFA training, these guidelines were made available online for the public to access. The guidelines were accessible through the National Health and Medical Research Council (NHMRC) Clinical Practice Guidelines Portal, and were also made available for free download from the MHFA website (https://mhfa.com.au/cms/guidelines). A study by Hart and colleagues [21] showed that users who download the guidelines do make use of them to assist in mental health first aid situations.

To ensure the guidelines reflect current evidence and best practice, re-development of these guidelines is required to update their content, to take into account the latest suicide prevention research findings and recommendations from suicide prevention experts. Re-development of the guidelines will also ensure that they meet the NHMRC Clinical Practice Guidelines Portal inclusion requirements, which require that guidelines be no more than five years old.

The aim of this study was to use the Delphi methodology [22] to re-develop guidelines for members of the public providing first aid assistance to people who are having suicidal thoughts or displaying suicidal behaviour. This method has been used to develop mental health first aid guidelines for a range of mental disorders, including the original version of the suicide guidelines. This method was selected as it is considered a feasible and ethical approach to developing guidelines on a topic that is not amenable to evaluation in randomised controlled trials. The method allows the gathering of practice-based evidence from experts, so that their expertise can be conveyed to others. The method also allows expert consensus from panel members located in many countries to be obtained easily online.

## Method

The re-development of the guidelines was conducted in three stages: literature search, questionnaire development and Delphi consensus survey rounds.

### Literature search

A systematic literature search was conducted to find statements about how someone can help a person who is suicidal, including how to determine if someone is having thoughts of suicide, how to offer short-term assistance to the person, and how to seek appropriate professional support for them. The literature searched included online materials, research publications, books and existing suicide intervention courses.

Websites and online materials were searched using the Google search engines of English-speaking countries (Google.com, Google.com.au, Google.co.uk, Google.nz, Google.ca). The search terms ‘suicide’ , ‘help’ (truncated to include terms such as ‘helping’ and ‘helped’) and ‘friend’ or ‘family’ were entered. The terms ‘survivor’ , ‘after suicide’ , ‘grief support’ , ‘aftermath’ and ‘bereave’ (truncated to include terms such as ‘bereavement’ and ‘bereaved’) were excluded to ensure the return of the most relevant sites. The websites returned in the top 50 results from each search were reviewed. Overall, 205 unique websites were reviewed for potential first aid helping actions, with relevant statements found on 66 of these sites.

The research literature was searched through PsycInfo and PubMed, with the terms ‘suicidal’ or ‘suicide’ and ‘help’ (truncated as above), as well as ‘prevent’ or ‘assist’ searched for in the title and abstract, and the exclusion of results containing the terms ‘cell suicide’ , ‘assisted suicide’ , ‘suicide attack’ and ‘homicide suicide’ to improve relevance of results. Articles published before 2004 were also excluded from the searches, as the searches aimed to find new articles that have not been covered by the literature search for the initial version of the guidelines. Searches on both these databases returned 853 articles, which were then screened for relevance. Following the screening process, 4 articles were deemed relevant. The irrelevant articles were excluded through a hierarchical screening process, starting with titles (n = 791), abstracts (n = 45) and then full-text (n = 13).

To locate relevant books, a search of Amazon.com was also conducted using the search terms ‘suicidal’ , ‘help’ and ‘friend’. Forty-five books were returned, with 5 of these considered relevant. These 5 books were purchased and read, with all containing relevant helping statements (references provided in Table 1).

**Table 1:** **List of original sources for statements that were included in the Round 1 questionnaire**

**Websites (N = 66)**	
HelpGuide.org	http://www.helpguide.org/mental/suicide_prevention.htm
Stop A Suicide	http://www.stopasuicide.org/signs.html
MayoClinic	http://www.mayoclinic.com/health/suicide/MH00058
ReachOut Australia	http://au.reachout.com/My-friend-is-suicidal
Suicide Prevention	http://www.suicideprevention.com.au/
Psych Central	http://psychcentral.com/lib/what-to-do-when-you-think-someone-is-suicidal/0007461
Better Health Victoria	http://www.betterhealth.vic.gov.au/bhcv2/bhcarticles.nsf/pages/Suicide_family_and_friends
NASP Resources	http://www.nasponline.org/resources/crisis_safety/suicideprevention.aspx
American Academy of Child and Adolescent Psychiatry	http://www.aacap.org/cs/root/facts_for_families/teen_suicide
Depression and Bipolar Support Alliance	http://www.dbsalliance.org/site/PageServer?pagename=help_crisis
Kids Help Line	http://www.kidshelp.com.au/teens/get-info/hot-topics/lets-talk-about-suicide.php
Befrienders Worldwide	http://www.befrienders.org/support/index.asp?PageURL=helpAfriend.php
Child and Youth Health South Australia	http://www.cyh.com/HealthTopics/HealthTopicDetails.aspx?p=240&np=29&&id=2061
University of Minnesota	http://www.extension.umn.edu/distribution/youthdevelopment/da2787.html
Lifeline	http://www.lifeline.org.au/Get-Help/Facts---Information/Preventing-Suicide/Preventing-Suicide
SANE	http://www.sane.org/information/factsheets-podcasts/434-sane-steps-how-to-help-when-someone-is-suicidal
Know the Signs	http://www.suicideispreventable.org/
University of Notre Dame Counselling Centre	http://ucc.nd.edu/self-help/depression-suicide/helping-someone-in-a-suicidal-crisis/
Survivors of Suicide	http://www.survivorsofsuicide.com/faq_suicide.shtml#7
Re Think	http://www.rethink.org/living_with_mental_illness/coping_in_a_crisis/suicide_self_harm/suicide/helping_a_suicidal_f.html
San Francisco Suicide Prevention	http://www.sfsuicide.org/prevention-strategies/how-to-help-someone/
U Matter	http://www.umatterucangethelp.com/
The University of North Carolina Campus Health Services	http://campushealth.unc.edu/services/counseling-and-psychological-services/how-support-student/concern-about-friend
American Foundation for Suicide Prevention	http://www.afsp.org/
Gustavus Alophus College Suicide Prevention Information	https://gustavus.edu/counseling/suicide-prevention.php
Real Warriors Military Support	http://www.realwarriors.net/family/support/preventsuicide.php
National Suicide Prevention Lifeline	http://www.suicidepreventionlifeline.org/Learn/WarningSigns
WikiHow	http://www.wikihow.com/Help-Someone-Who-Is-Thinking-About-Committing-Suicide
Suicide Call back Service	http://www.suicidecallbackservice.org.au/concerned-about-someone/index
WebMD	http://www.webmd.com/depression/depression-suicide-signs
University of Illinois Counselling Center	http://www.counselingcenter.illinois.edu/?page_id=148
Choices: National Health Service UK	http://www.nhs.uk/Conditions/Suicide/Pages/helping-others.aspx
Quebec Health Portal	http://www.msss.gouv.qc.ca/sujets/prob_sante/sante_mentale/index.php?suicide_en
Mend a Friend	http://www.mend-a-friend.com/speaking-to-a-distressed-friend/types-of-questions/
Queensland Government	http://www.qld.gov.au/youth/health-looking-after-yourself/suicide/
Suicide: It’s no secret	http://nosecret.org.au/how_do_i_help_my_friends
World Suicide Prevention Day: Suicide Prevention Australia	http://wspd.org.au/help/
Schizophrenia Fellowship of New South Wales	http://www.sfnsw.org.au/Carer/FAQs/What-Should-I-do-If-Someone-Is-Suicidal/default.aspx
FNQ Suicide Prevention Taskforce	http://www.suicidepreventionfnq.org.au/prevention&intervention.html
Grapevine Group	http://www.grapevinegroup.org.au/help.php
Super Friend	http://www.superfriend.com.au/individuals/get-the-facts/suicide-and- bereavement/how-to-help-when-someone-is-suicidal
Queensland Government Health	http://access.health.qld.gov.au/hid/ChildHealth/MentalHealth/suicidePrevention_is.asp
Society for the Prevention of Teen Suicide	http://www.sptsusa.org/parents/friend-of-child.html
Erase Bullying	http://www.erasebullying.ca/youth/youth-suicide-want.php
Crisis Centre	http://www.crisiscentre.bc.ca/get-help/understanding-and-recognizing-suicide/#responding
Here to Help	http://www.heretohelp.bc.ca/factsheet/suicide
Health Link	http://www.healthlinkbc.ca/kb/content/symptom/suicd.html#hw111129
New Brunswick Canada Health	http://www.gnb.ca/0055/help-e.asp
Government of Alberta Information for teens	http://www.edmontonandareacfsa.gov.ab.ca/publish/551.cfm
Half of Us	http://www.halofus.com/learn-the-signs/
National Alliance on Mental Illness	http://www.nami.org/Template.cfm?Section=By_Illness&Template=/ContentManagement/ContentDisplay.cfm&ContentID=23041
Mental Health America	http://www.mentalhealthamerica.net/go/suicide
The Kelty Foundation	http://www.thekeltyfoundation.org/resources.php
CHEO Health Center	http://www.cheo.on.ca/en/suicideinfo
Alberta Health Services	https://myhealth.alberta.ca/health/pages/conditions.aspx?hwid=suicd#hw111129
Western University Health and Wellness	http://www.shs.uwo.ca/mentalhealth/depression_other/suicide.html
C-Health	http://chealth.canoe.ca/channel_condition_info_details.asp?disease_id=135&channel_id=40&relation_id=55627
Faze Youth Magazine	http://www.faze.ca/issue03/preventing_suicide.html
Youth Services Jeunesse	http://www.ysb.on.ca/index.php?page=know-what-to-do-preventing-youth-suicide&hl=eng
Crisis Outreach and Support Team Hamilton	http://coasthamilton.ca/?page_id=100
Suicide Prevention Information New Zealand	http://www.spinz.org.nz/page/298-responding-to-people-at-risk+how-can-you-help-someone-who-is-at-risk
New Zealand Ministry of Health	http://www.health.govt.nz/yourhealth-topics/mental-health/suicide-prevention
Massey University New Zealand Health and Counselling Services	http://www.massey.ac.nz/massey/student-life/services-and-resources/health-counselling-services/resources/self-injury--suicide/suicide.cfm
When Your Head Spins	http://www.wordworx.co.nz/help.html
Life	http://www.life.org.nz/suicide/suicidefaq/suicidefaq14/
Kids Help Phone	http://org.kidshelpphone.ca/en/suicide
Mental Health First Aid Australia	https://mhfa.com.au/cms/guidelines
Books (N = 5)	
Hill K, Gorman J: How to help someone who is suicidal. Mind Publications 1995	
Marcus, E: Why suicide? Questions and answers about suicide, suicide prevention, and coping with the suicide of someone you know. HarperCollins 2013	
Gordon, S: When living hurts: What-to-do book for yourself or someone you care about who feels discouraged, sad, lonely, hopeless, angry or frustrated, unhappy, bored, depressed, suicidal. URJ Press 2004	
Nelson, RE: The power to prevent suicide: A guide for teens helping teens. ReadHowYouWant.com 2009	
Cook J: How to help someone who is depressed or suicidal: Practical suggestions from a survivor. Rubicon Press Inc. 1993	
Suicide prevention course materials (N = 6)	
*ASIST* (Livingworks)	
*SafeTalk (Livingworks)*	
*Suicide Prevention Skills Training (Griffith University)*	
*ACE* (The US Military)	
ASK about Suicide (The University of Texas)	
*QPR* (The Salvation Army, Australia)	
Journal articles (N = 4)	
Barrero SA: Preventing suicide: A resource for the family. *Annals of General Psychiatry* 2008, 7:1	
Deisenhammer EA, Ing CM, Strauss R, Kemmler G, Hartmann H, Weiss EM: The duration of the suicidal process: How much time is left for intervention between consideration and accomplishment of a suicide attempt? *Journal of Clinical Psychiatry* 2009, 70:1	
Kelly CM, Jorm AF, Kitchener BA, Langlands RL: Development of mental health first aid guidelines for deliberate non-suicidal self-injury: A Delphi study. *BMC Psychiatry* 2008, 8:62	
Norris D, Clark MS: Evaluation and treatment of the suicidal patient. *American Family Physician* 2012, 85:6	

Existing suicide intervention course materials were also obtained where possible. This involved searching for courses online, contacting the organisations and requesting course materials where these were not available online. We were able to locate course materials from 6 suicide prevention courses, as listed in Table 1. Materials obtained included participant workbooks and trainer notes, all of which were thoroughly read, with relevant helping statements extracted.

### Questionnaire development

Relevant helping statements that were found in the literature search, as well as the statements included in the previous Delphi questionnaires, formed the content of the first questionnaire. Statements were considered acceptable for inclusion in the questionnaire if all three authors agreed that they described how someone can help a person who is suicidal with clear and non-ambiguous actions. For example, the statement ‘Talk to the suicidal person in a private place’ was considered acceptable, as it clearly specifies what actions are required by the first aider. The statement ‘Try to connect with the suicidal person’ was considered unacceptable, as it does not specify the actions the first aider should take or what is meant by the term ‘connect’.

These statements were grouped into categories based on common thematic content. Statements were edited so that those with similar content were combined to reduce repetition throughout the questionnaire. Statements were also edited to improve clarity, through systematic re-wording or elaboration through examples. This editing occurred in meetings of the working group, which were held to edit and develop a draft of the questionnaire, including its categories and structure of statements. The working group comprised the authors of this paper who are all researchers with previous experience in conducting research using the Delphi methodology and in MHFA training programmes.

The questionnaire was completed online through an online survey website, Survey Monkey. Participants were given a two to three week time period to finish the questionnaire for each of the three rounds. The questionnaires could be completed at times that were convenient to participants, and in multiple sittings if desired.

### Delphi consensus survey rounds

The consensus survey was conducted using the Delphi method. (Jones et al., [22]). The Delphi method involved identifying and recruiting panels of experts in the field of suicide prevention to rate the importance of helping statements. Statements that achieved substantial consensus regarding their importance for inclusion in the guidelines were considered as the recommended actions to help someone who is experiencing suicidal thoughts.

Participants were recruited from developed English-speaking countries (Australia, United Kingdom, Ireland, Canada, United States and New Zealand) to join one of two expert panels representing two areas of expertise: professionals or consumers. To be considered as having expertise in suicide prevention, panellists were required to have professional experience working in the field of suicide prevention (i.e. as a researcher, clinician, mental health worker, social worker), or personal experience with suicidal thoughts and/or attempts. Potential professional panellists were identified as experts through their involvement with suicide prevention organisations, while potential consumer panellists through their advocacy roles in suicide prevention.

The professional panel comprised 41 experts, some of whom had multiple roles, including 11 professors and 7 associate professors in psychiatry or psychology, 9 psychologists, 8 psychiatrists, 3 mental health nurses, 3 suicide prevention researchers, 2 suicide support program coordinators, 2 physicians, 1 social worker, and 3 who worked in other mental health support roles. This panel represented global professional opinions in suicide prevention, coming from many different English-speaking backgrounds (see Table 2). Professional panellists were recruited through editorial boards of relevant academic journals and suicide prevention organisations. The heads of these boards and organisations were emailed an invitation to participate and a copy of the project’s plain language statement, asking these to be forwarded on to the relevant members. The academic journal editorial boards contacted included *Crisis* and *Suicide and Suicidal Behaviour*. Professional panellists were also recruited through suicide prevention organisations, such as the International Association of Suicide Prevention, Suicide Prevention Australia, the Australian Suicide Prevention Advisory Council, the American Foundation for Suicide Prevention, the American Association of Suicidology, the Canadian Association for Suicide Prevention, the Suicide Prevention Resource Center, the University of Oxford Centre for Suicide Research and Suicide Prevention Information New Zealand. Professionals were also asked to nominate any colleagues who they felt would also be appropriate panel members.

**Table 2:** **Participant characteristics (data collected in Round 1)**

	**Age range**	**Median age**	**% Female**	**Americans**	**Australians**	**Europeans**	**Canadians**
Mental health professionals (n = 41 )	28-71	50	32	13	12	9	5
Consumers (n = 35 )	24-66	47	77	9	22	0	4

The consumer panel comprised of 35 suicide consumer advocates (people who have experienced suicidal ideation or made a suicide attempt in the past). Consumers were recruited through depression and mental disorder advocacy organisations, including beyondblue (Australia), Depression and Bipolar Support Association (United States), National Alliance of Mental Illness (NAMI) (United States), Depression Alliance (United Kingdom), and Depression Support Network (New Zealand). In a similar fashion to recruitment of the professional panel, email invitations and plain language statements were emailed to the advocacy group coordinators for the information to be forwarded on to the group members. Consumers who had written websites that offered support and information to other consumers, as well as promoted recovery from suicidal ideation, were also identified as potential panellists. Considered as online advocates, they were also invited to participate through email invitation. Consumers were also asked to nominate anyone they knew who they felt would also be appropriate panel members.

The outcome for each item was determined using predetermined criteria. Statements that were rated as essential or important by 80% or more of the members in both panels were endorsed as helping actions to be included into the guidelines. Statements were re-rated in a subsequent round of the questionnaire if they were rated as essential or important by 70–79.9% both of the panels, or they were rated as essential or important by 80% of more of one panel, but less than 80% by the other panel. Statements that were rated as essential or important by less than 70% of both panel members were excluded.

Participants were instructed to rate the statements presented in each questionnaire according to how important they believed they are to the aims of mental health first aid and the role of the first aider (detailed participant instructions are included in the Additional files 1, 2 and 3). In Round 1, panel members were also asked to provide feedback through a textbox at the end of each section of the questionnaire. This feedback textbox was intended for use by panellists to suggest helping actions that were not covered in the questionnaire, but generally panellists used the textboxes to provide rationales for their ratings. The comments made were reviewed by the working group. Suggestions that contained novel ideas were used to create new helping statements to be included in the subsequent Round 2 questionnaire. Also, statements that received feedback suggesting ambiguity in the interpretation of its meaning were re-phrased to make them clearer and included in Round 2. Statements from Round 1 that met the criteria to be re-rated were also included in the Round 2 questionnaire.

The third and final questionnaire was comprised of new statements that were developed from Round 1 feedback and presented for the first time in Round 2, but required re-rating in a further round. Items that still did not achieve consensus after being re-rated were rejected from inclusion in the guidelines.

Following each of the three rounds, each panellist was sent a report containing a summary of the results from the previous round. The report included a list of the statements that had been endorsed for inclusion in the guidelines, as well as a list of the statements that had been rejected from inclusion. The statements to be re-rated in the subsequent round were also included, with the report personalised to include the individual panellist’s rating for each statement, as well as a table summary of each panel’s ratings for the statement.

The statements that were endorsed across the three survey rounds were compiled. These statements were then used to form the guidelines, with working group meetings held to finalise structure and wording. The final draft copy of the guidelines was then disseminated to panellists for their final comment on the document. While panellists could not suggest new content at this stage, they were able to provide feedback on the wording of the document to improve clarity and reduce ambiguity.

## Results

Participant characteristics are included in Table 2, with participation of suicide prevention professionals and consumer advocate panellists across the three Delphi survey rounds shown in Table 3. The section headings of the Delphi questionnaire that the items were categorised into are shown in Table 4, as is the number of items that were endorsed and rejected in these sections.

**Table 3:** **Participation of Delphi panellists in each round**

	**Round 1**	**Round 2**	**Round 3**	**Retention rate (over 3 rounds)**
Suicide prevention professionals	41	32	27	65.9%
Consumers	35	23	21	60.0%

**Table 4:** **Sections in the Delphi questionnaire, number of items endorsed and rejected, and correlations between panel ratings for each section**

**Section**	**Topic**	**Number of items endorsed**	**Number of items rejected**	**Correlation between panel ratings (Pearson’s r)**					
				**Round 1**			**Round 2**		
				**r**	**df**	**t**	**r**	**df**	**t**
1	Identification of suicide risk	28	24	0.86*	46	11.31	0.89*	15	7.22*
2	Assessing seriousness of risk	16	16	0.92*	24	11.42	0.64*	12	2.78
3	Initial assistance	31	69	0.92*	101	22.83	0.42*	22	2.12*
4	Talking with the suicidal person	39	37	0.94*	74	23.76*	-		
5	No-suicide contracts	8	18	0.92*	25	11.34	-		
6	Ensuring safety	0	9	0.95*	8	9.07	-		
7	Passing time during the crisis	0	13	0.90*	12	8.53	-		
8	What the first aider should know	15	7	0.66*	19	4.24	-		
9	Confidentiality	7	5	0.98*	11	19.42	-		
10	Adolescent-specific	20	72	0.88*	91	18.32	0.85*	16	6.19

Note: Correlations are not reported for sections where there were less than 10 items rated. Correlations for Round 3 have not been provided as there were not enough ratings to determine a valid correlation coefficient. *p < .05.

Pearson’s r was calculated to determine the correlations between the professional and consumer panels’ ratings. For the 416 items rated in Round 1, and the 96 items in Round 2, the item endorsement rates from the consumer panel and the professional panel were strongly correlated, with correlation coefficients of .92 (*t*(415) = 47.82, *p* < .05) and .80 (*t*(101) = 13.40, *p* < .05) for these rounds respectively. Correlations between the panels’ ratings for each section are also shown in Table 4.

**Figure 1:**
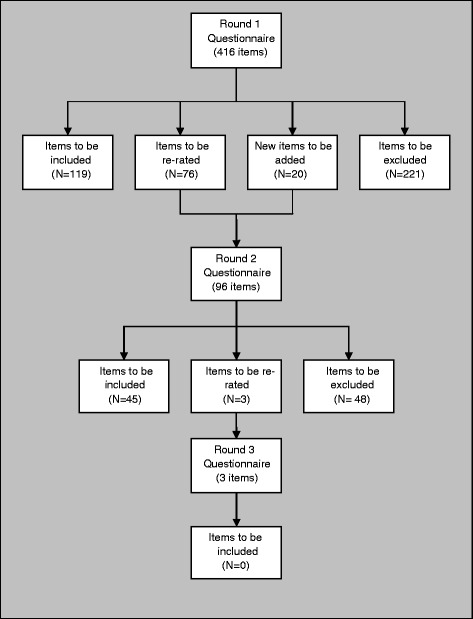
**Overview of statements throughout the 3 rounds of questionnaires.**

The inclusion, exclusion and re-rating rates for each round are shown in Figure 1. The 164 statements that were endorsed for inclusion in the guidelines for suicidal thoughts and/or behaviours are can be viewed in Additional file 4 Table S1. These statements are the helping actions a member of the public should be guided by to provide first aid to someone who is experiencing suicidal thoughts, or engaging in suicidal behaviour. These statements were then incorporated into a plain language document to comprise the guidelines (see additional file).

## Discussion

The aim of this study was to re-develop first aid guidelines for members of the public in providing assistance to someone experiencing suicidal thoughts or engaging in suicidal behaviours. This was achieved by searching the available literature for recommended helping actions, with expert panellists, then rating their importance for inclusion in the guidelines. Statements that achieved high endorsement consensus across both panels were included in the re-developed guidelines.

Statements that were endorsed for inclusion in the guidelines promote the first aider connecting with the person directly, through discussion and providing support, as well as connecting the person with additional social support, such as family members and close friends, and professional support. The first aider is more likely to be part of the person’s family or circle of friends who are important to sense of belongingness, and their actions can increase the sense of belongingness in a way that a professional cannot. As proposed by the interpersonal theory of suicide [6], increasing the sense of social connectedness and belongingness can reduce the desire to suicide and therefore the risk of carrying out suicidal behaviour.

### Comparison with original guidelines for suicidal thoughts and behaviours

In comparison with the original 2008 version of the guidelines, some significant similarities and differences can be noted. For the 94 statements that appeared in both the current and the 2008 Delphi, the endorsement ratings given by each panel were quite similar. The endorsement rates for statements given by the current panel were found to correlate with those given in the 2008 study, with a Pearson’s correlation of .84 (*t*(93) = 14.93, *p* < .05) between the professional panels and .77 (*t*(93) = 11.64, *p* < .05) between the consumer panels.

Similarities were also seen when examining specific sections of the earlier and current guidelines. In both versions of the guidelines, no items were endorsed in the ‘Ensuring safety’ section, which involved removing harmful items and means for suicide from the suicidal person, and the ‘Passing time during the crisis’ section, which consisted of activities and or distractions the first aider should do with the suicidal person until the strong urge for suicide passes. This suggests that expert opinion in these areas has not changed greatly over the past 6 years, with consensus not reached regarding when and how first aiders should remove suicide means, and first aiders not recommended to use distractions during a suicide crisis.

However, there were some important differences from the earlier guidelines. The re-developed guidelines provide a more comprehensive set of first aid actions than those developed by Kelly et al. [13]. The current Delphi survey comprised 436 novel statements that were rated by the panellists over the three rounds. This is a substantial 322 more statements than the 114 novel statements that comprised the original Delphi questionnaires. Compared with the 30 originally endorsed statements, the redevelopment of the guidelines saw 164 statements endorsed. Of these, 24 items were re-endorsed from the original guidelines. The substantial increase in endorsed statements makes the recommended helping actions more specific and detailed, reducing uncertainty around how to carry out an action through being more directive.

More detail is particularly notable throughout the sections of the guidelines outlining identification of suicide risk, provision of initial assistance and talking with the suicidal person, giving more specific information as to when, where and how actions should be carried out. Over 20 extra statements were endorsed for each of these sections compared with the original guidelines. These statements include more specific actions that a first aider can take how to talk to the suicidal person, what to talk about with the suicidal person, things to avoid saying to the suicidal person, and what to include when developing a safety plan.

Furthermore, the ‘Initial Assistance’ section had 33 statements endorsed regarding how the first aider should provide assistance in the first instance. This is a marked increase on the 2 items endorsed in the same section in the original guidelines, which was of particular concern to the authors. The endorsement of only two statements in this section was not considered to provide sufficient guidance to the first aider, and to compensate for this, the authors wrote a paragraph acknowledging the importance of involving professional help. The re-developed guidelines now provide a great deal more direction in how and when to engage professional help, and the appropriate helping professionals to contact depending on the urgency of suicide risk (i.e. who to contact if the person is having thoughts of suicide compared with who to contact if the suicidal person has both a plan and the means to carry out their plan).

The re-development of the guidelines also saw the addition of two questionnaire sections; ‘adolescent-specific’ and ‘what the first aider should know’. The inclusion of adolescent-specific statements provided recognition that suicidal adolescents may need more guidance and support compared to a suicidal adult. This would allow for the person providing the first aid to tailor their assistance in an age-appropriate manner. However, only two differences were noted in the ratings of initial assistance statements endorsed for adolescents compared to adults. These involved the first aider making sure someone close to the suicidal adolescent knows about the situation if the adolescent is reluctant to seek help, and the first aider getting assistance from a mental health professional if the adolescent refuses professional help. The other additional section outlined what information the first aider should know to place them in the best position to provide assistance. This included knowledge of the warning signs and risk factors for suicide, as well as clarification of the myths and the facts about suicide.

This increase in both the number of and detail in the recommendations included in the guidelines can be considered a reflection of the growth of advice available on the internet, as most items in the Delphi questionnaire were generated from web-based sources over the past 6 years since the development of the original guidelines. This increase also indicates an increase in suicide prevention expertise and research literature over the past 6 years. This highlights the importance of conducting revisions of guideline documents, as much change in the literature and expert opinion can occur across the span of a few years.

A further difference is that carers of persons who had been suicidal were included as expert panellists for the development of the 2008 guidelines, but were not used as experts in the re-development. Typically in MHFA guideline development, three expert panels have been recruited; mental health professionals, consumers and carers. Kelly et al. [13] tried to represent the expertise and experiences of those who have cared for suicidal persons, but found that carers were difficult to recruit. There were not enough carers to form their own panel, which resulted in their endorsements being combined with those of the consumer panel in the end. Due to these difficulties, the working group made the decision to not have a carer panel in this Delphi study.

### Comparison between ratings of professional and consumer panels

Overall, professionals and consumers rated items similarly, with high correlations between the panels’ ratings. This indicates that both panels had similar priorities in the re-developed guidelines, generally agreeing on what helping actions should be included and what should not. This included agreement on the importance of the first aider acting promptly to ask about suicidal thoughts, and knowing the myths and facts about suicide. Both professionals and consumers also agreed on the importance of the first aider connecting the suicidal person with professional help, as well as interacting with the person in an understanding, empathic and calm manner.

However, while the ratings were quite similar, some notable differences were evident in the ratings assigned to statements between the professional and consumer panels. As could be expected, consumer ratings tended to emphasise actions that provide a caring and understanding experience for the suicidal person, having much consideration for the suicidal person’s feelings and experiences. For example, 85% of consumers highly endorsed that ‘The first aider should keep in mind that asking too many questions can provoke anxiety in the suicidal person’, whereas the professional panel did not endorse this statement, with only 69% rating it as either important or essential. The consumer panel also gave higher endorsement ratings to statements related to providing reassurance and support to the suicidal person. For example, 76% of consumers endorsed the statement ‘Remind the suicidal person that they are loved and would be missed’ while only 33% of professionals rated it as essential or important.

On the other hand, professionals assigned higher ratings to statements that involved the first aider gathering information about the suicidal person’s situation, which places the first aider in a position to make informed decisions about what to do next. This included giving higher ratings to statements that involved the first aider engaging in active listening, being aware of the myths and facts about suicide, validating the person’s problems and their thoughts of suicide, and gathering information about the urgency of suicide. For example, the statement ‘Ask the suicidal person how they intend to suicide i.e. ask them direct questions about how, when and where they intend to suicide’ was highly endorsed by 95% of professionals, with only 76% of consumers giving it a rating of important or essential in Round 1. Similarly in Round 1, 92% of professionals compared to 79% of consumers endorsed the statement ‘Ask the suicidal person if they have ever made a suicide plan in the past’. Furthermore, 83% of the professional panel, but only 64% of the consumer panel, endorsed the statement ‘The first aider should know that suicidal thoughts are temporary’.

### Strengths

The most important strength of this study is that it has ensured that the guidelines contain the most current and up-to-date recommendations, reflecting the most recent recommendations in the suicide prevention literature. In doing so, the current guidelines provide greater depth and direction to guide the administration of first aid than the 2008 guidelines. Furthermore, the larger panel sizes recruited for the guideline re-development give more stable results than those obtained in the previous Delphi study. Compared with the original guidelines, 38 more panellists participated in the first round questionnaire. This increase in panel numbers indicates that a much broader range of suicide prevention expertise and experiences was drawn upon in the guideline re-development.

### Weaknesses

Despite recruiting the recommended panel sizes, there were drop-outs across the rounds of the study. Only 68% of consumers participated in all three questionnaire rounds, with 76% of professionals taking part in all three rounds. As the first survey was expected to take approximately 1 hour to complete, the time commitment required for the first round questionnaire may have deterred panellists from participation in subsequent rounds. However, despite these drop-out rates, the recommendation of a minimum of 23 Delphi panellists [22] was reached for both panels for the rating of the majority of items which occurred in Round 1.

Furthermore, while these guidelines have included adolescent-specific statements to allow first aiders to tailor their assistance in a developmentally appropriate manner, these guidelines have not been developed to incorporate cultural differences. The application of the guidelines to non-western cultures and ethnic minorities is an area requiring further investigation and consultation with suicide prevention experts from these cultural and ethnic backgrounds.

The use of these guidelines is recommended for use by first aiders only. While the actions endorsed in these guidelines may be useful in different aspects of the suicide prevention continuum, from preventing the onset of suicidal ideation itself to supporting the suicidal person in a professional setting, these are specific to the recommended support that can be provided by a first aider. These guidelines take into consideration the limitations in the first aiders’ support role, and guide the first aider on how to act within these.

Finally, it must be kept in mind that the helping actions endorsed in the guidelines are based on expert opinion, and that these are the recommendations of experts and have not been generated from an empirical study. Also, the endorsement of more helping actions adds greater complexity to the guidelines and their implementation. This increase in complexity could possibly be a barrier for implementation for some first aiders.

## Conclusion

Through the Delphi process, the first aid guidelines for suicide have been updated to ensure they are current and include the most recent and appropriate helping actions. This re-development has added depth to the previous version of the guidelines, giving more guidance in providing initial assistance, involving mental health professionals, talking with the suicidal person, and providing assistance to suicidal adolescents, as well as important background information and facts about suicide. These guidelines will now be made freely available for download on the MHFA website, will be used to update the MHFA course, and will also used to form the basis of a suicide prevention course aimed at educating members of the public in providing first aid to someone who is experiencing suicidal thoughts.

## Additional files

Additional file 1:
**Round 1 questionnaire.**
Additional file 2:
**Round 2 questionnaire.**
Additional file 3:
**Round 3 questionnaire.**
Additional file 4:
**Items endorsed into and rejected from the guidelines, endorsement ratings across the three rounds and the corresponding round consensus was reached.**
