# Supplementary material for: Re-development of mental health first aid guidelines for suicidal ideation and behaviour: a Delphi study
Source: BMC Psychiatry. 2014 Sep 13;14:241. doi: 10.1186/s12888-014-0241-8 (PMC4199061; doi:10.1186/s12888-014-0241-8)
Supplement: Additional file 1: — Round 1 questionnaire. [file 12888_2014_241_MOESM1_ESM.pdf]

Redevelopment of mental health first aid guidelines for suicidal thoughts

Introduction

Purpose of the research

The aim of this project is to re-develop guidelines for members of the public providing first aid for people who are having suicidal thoughts or displaying suicidal behaviour. This redevelopment will ensure the guidelines remain consistent with the current literature and contain the most recent and appropriate helping actions, as well as continue to meet the standards for their inclusion on the NHMRC Clinical Practice Guidelines Portal.

As a first step, we are seeking to find current consensus on the key skills and knowledge that a person needs to help someone who is having suicidal thoughts or displaying suicidal behaviour. The statements from this questionnaire that receive a high level of consensus will be included in the mental health first aid guidelines for suicidal thoughts. The guidelines will then be used as the basis for an intervention training program.

Your role

You have been selected as a panel member for this study because you have expertise in the area of suicide prevention. Your task is to rate the statements presented in this questionnaire according to how important you believe they are to the aims of mental health first aid and the role of the first aider.

The role of a Mental Health First Aider is to help a person who is developing a mental health problem or is in a mental health crisis. The first aid is given until appropriate professional treatment is received or the crisis resolves.

How this questionnaire was developed

The statements in the following questionnaire were derived from suicide prevention course materials, published help books, websites and online fact sheets. Some of the statements may seem contradictory or controversial; however, we have included them because they reflect the wide range of people's beliefs about intervention and care.

It is important to note that we do not necessarily agree with these statements; we have included them because we do not believe that we should decide what the best practice is for mental health first aid. Rather, we have invited you to be a member of the expert panel to help develop a set of guidelines that reflect current expert opinion across the field of suicide prevention.

Other instructions to note

This questionnaire should take approximately 60 minutes to complete. However, some people may finish it much more quickly. Others may take more time, especially if they wish to lodge comments at the end of each section. Please be aware that once you have logged on and started responding, you may save your answers at any time by completing a page and clicking "Next" at the bottom. This marks your page as complete and you may begin again another time at the next page. Please make sure that you always log back in using the same email link, otherwise the software will fail to recognise your code and previously saved responses.

We would like to **thank you** for your time and effort and encourage you to provide us with feedback on this process.

Best wishes,

The Research Team
Population Mental Health Group
Melbourne School of Population and Global Health
University of Melbourne

Definitions of terms used in this questionnaire

Throughout the questionnaire, we use the terms **'the person'** to refer to the person experiencing suicidal thoughts or engaging in non-suicidal self-injury, and **'the first aider'** to refer to the person providing assistance.

Mental health problem is a broad term that includes developing mental illness, symptoms of a diagnosable illness, substance use, and adverse life events which are having an impact on functioning. A **mental illness** is a mental health problem that has been diagnosed by a mental health professional, that affects a person's thinking, emotional state and behaviour, and disrupts a person's ability to work or carry out other daily activities and engage in satisfying personal relationships.

Emergency services refers to the best services available at short notice for the situation in the area. **Emergency mental health services** refers to mental health services that are available at short notice in the area, and could include a crisis team or a suicide helpline.

A **Safety plan** is an agreement between the suicidal person and the first aider that involves actions to keep the suicidal person safe.

Please be aware that the statements in this survey only apply to first aid given by adults. However, there are some statements specifically about the support that an adult could provide to an adolescent. These items are included in cases where the support provided to an adolescent might be quite different to the support provided to an adult. The term **adolescent** refers to a young person aged between 12 and 18 years (nominally the high school years).

Overview of suicide questionnaire content

Please note that there will be an opportunity to add any **comments or suggestions** you might have at the **end of each section** of the survey.

Section 1- Identification of suicide risk

• *Things to consider before making an approach*
• *How to approach the person of concern*
• *Asking about suicidal thoughts*
• *What to do if the person of concern doesn't want to talk*
• *What to do if the first aider feels unsuccessful in their approach*
• *What to do if the person of concern is psychotic*
• *What to do if the person of concern is under the influence of drugs and alcohol*

Section 2- Assessing seriousness of the suicide risk

- *Assessing the urgency of the risk*
- *Finding out about the suicidal person's plan*
- *Asking about the factors that contribute to risk*
- *Asking about other factors that contribute to risk*

Section 3- Initial assistance

- *Providing initial assistance to the suicidal person*

Section 4- Talking with the suicidal person

- *What to do when talking with the suicidal person*
- *Things to avoid when talking with the suicidal person*

Section 5- No-suicide contracts

- *Developing a safety plan*
- *What the safety plan should include*
- *Timeframe of the safety plan*
- *When to make a safety plan*

Section 6- Ensuring safety

- *Reducing the chances of the suicidal person acting on suicidal thoughts*

Section 7- Passing time during the crisis

- *What to do until the suicide crisis passes*

Section 8- What the first aider should know

- *The facts about suicide*
- *The preventable nature of suicide*

Section 9- Confidentiality

- *Determining what information needs to be shared with others*

Section 10- Adolescent specific

- *Helping statements that may differ in endorsement for people aged 12-18 compared to adults*

Demographic and mental health role information

Please provide your details below to help us with our research

Your gender

○ Male ○ Female

Your age (in years)

[ ]

Which best describes your area of expertise?

- ◯ Suicide prevention professional
- ◯ Suicide consumer advocate

What is your occupation and title?

Please name all the mental health organisations you are affiliated with and your role within each organisation.

Please provide details of your primary place of work

City/Town

State

Country

Section 1. Identification of suicide risk

This section contains statements about identifying the severity of the person's risk of suicide.

NOTE: If the person has already acted on their thoughts of suicide, this should be treated as a medical emergency.

Please rate how important (from *essential* to *should not be included*) you think it is that each statement be included in the final guidelines.

Please note that there will be an opportunity to add any **comments or suggestions** you might have at the **end of each section** of the survey.

Before approaching the person

1. The first aider should not assume that the person will get better without help.

Essential	Important	Don't know/Depends	Unimportant	Should not be included
◯	◯	◯	◯	◯

2. The first aider should not assume that the person will seek help on their own.

Essential	Important	Don't know/Depends	Unimportant	Should not be included
◯	◯	◯	◯	◯

3. The first aider should be able to recognise the warning signs of suicide.

Essential	Important	Don't know/Depends	Unimportant	Should not be included
◯	◯	◯	◯	◯

4. If the suicidal person has already harmed themselves, the first aider should administer first aid and call emergency services, asking for an ambulance.

Essential	Important	Don't know/Depends	Unimportant	Should not be included
◯	◯	◯	◯	◯

5. The first aider should talk to other people who know the person to see if they also have concerns.

Essential	Important	Don't know/Depends	Unimportant	Should not be included
◯	◯	◯	◯	◯

6. The first aider should have suicide crisis resources on hand before starting a conversation with someone who might be having suicidal thoughts.

Essential	Important	Don't know/Depends	Unimportant	Should not be included
◯	◯	◯	◯	◯

7. The first aider should act promptly if they think someone is considering suicide.

Essential	Important	Don't know/Depends	Unimportant	Should not be included
◯	◯	◯	◯	◯

8. The first aider should choose a private place to talk the person about their concerns.

Essential	Important	Don't know/Depends	Unimportant	Should not be included
◯	◯	◯	◯	◯

9. The first aider should choose a time to talk to the person when there is sufficient time to discuss their concerns.

Essential	Important	Don't know/Depends	Unimportant	Should not be included
◯	◯	◯	◯	◯

Section 1. Identification of suicide risk continued (1)

How to make the approach

The first aider should:

	Essential	Important	Don't know/Depends	Unimportant	Should not be included
10. Tell the person their concerns about them, describing behaviours that have caused them to be concerned about suicide.	○	○	○	○	○
11. Avoid raising the topic of suicide with the person during an argument or if they are really upset because this may end up getting a bad reaction and distancing them.	○	○	○	○	○
12. Be aware of their attitudes about suicide and the impact of these upon their ability to provide assistance, e.g. Beliefs that suicide is wrong or that it is a rational option.	○	○	○	○	○
13. Demonstrate appropriate language when referring to suicide by using the terms 'suicide' or 'died by suicide'.	○	○	○	○	○
14. Avoid using terms to describe suicide that promote stigmatising attitudes, e.g. 'commit suicide' or refer to a suicide attempt as having 'failed' or been 'unsuccessful'.	○	○	○	○	○
15. Be aware that different cultures have different beliefs and attitudes about suicide.	○	○	○	○	○

16. If the first aider is concerned about someone who is from a different cultural background to their own, they should learn about that culture's beliefs and attitudes towards suicide before approaching the person.

Essential	Important	Don't know/Depends	Unimportant	Should not be included
○	○	○	○	○

Asking about suicidal thoughts

17. If the first aider thinks someone might be having suicidal thoughts, they should ask that person directly.

Essential	Important	Don't know/Depends	Unimportant	Should not be included
○	○	○	○	○

18. The first aider should ask the person about suicidal thoughts, even if the first aider feels uncomfortable doing so.

Essential	Important	Don't know/Depends	Unimportant	Should not be included
○	○	○	○	○

19. If the first aider has even a mild suspicion that the person is having suicidal thoughts, they should ask.

Essential	Important	Don't know/Depends	Unimportant	Should not be included
○	○	○	○	○

20. If the first aider thinks someone might be having suicidal thoughts and feels unable to ask them, the first aider should find someone who is able to ask.

Essential	Important	Don't know/Depends	Unimportant	Should not be included
○	○	○	○	○

The first aider should:

	Essential	Important	Don't know/Depends	Unimportant	Should not be included
21. Not avoid using the word 'suicide'. It is important to discuss the issue directly, without dread or expressing negative judgement.	○	○	○	○	○
22. Know that it is more important to ask about suicidal thoughts than to be concerned about the exact wording.	○	○	○	○	○

The first aider should be aware that:

	Essential	Important	Don't know/Depends	Unimportant	Should not be included
23. If a person is not suicidal, asking them cannot put the idea of suicide in their head.	○	○	○	○	○
24. If a person is suicidal, asking them about suicidal thoughts will not increase the risk that they will act on these.	○	○	○	○	○
25. If a person is suicidal, asking them about suicidal thoughts will allow them the chance to talk about their problems and show them that somebody cares.	○	○	○	○	○

26. If the first aider thinks someone might be having suicidal thoughts, they should ask that person indirectly at first (i.e. "Do you ever wish you did not wake up in the morning?") and then only ask directly if the person says yes.

Essential	Important	Don't know/Depends	Unimportant	Should not be included
○	○	○	○	○

27. The first aider should ask the suicidal person if they know anyone who has suicided and if they are trying to solve their problems like they did.

Essential	Important	Don't know/Depends	Unimportant	Should not be included
○	○	○	○	○

28. If the first aider thinks someone might be having suicidal thoughts, they should begin the conversation by asking the person about how they are feeling.

Essential	Important	Don't know/Depends	Unimportant	Should not be included
○	○	○	○	○

29. The first aider should ask about and allow the person time to discuss their negative feelings before asking about suicidal thoughts.

Essential	Important	Don't know/Depends	Unimportant	Should not be included
○	○	○	○	○

Section 1. Identification of suicide risk continued (3)

If the person doesn't want to talk

30. The first aider should understand that the person may not want to talk with them, and should offer to help them find someone else to talk to.

Essential	Important	Don't know/Depends	Unimportant	Should not be included
○	○	○	○	○

31. If the first aider thinks the person is uncomfortable interacting with them due to differences in age group or gender, they should ask the person if they would prefer to talk to someone of the same age group or gender.

Essential	Important	Don't know/Depends	Unimportant	Should not be included
○	○	○	○	○

32. If the first aider thinks the person is uncomfortable interacting with them due to differences in cultural background, they should ask the person if they would prefer to talk to someone of the same cultural background.

Essential	Important	Don't know/Depends	Unimportant	Should not be included
○	○	○	○	○

If the first aider feels unsuccessful in their approach

33. If the first aider is unable to make a connection with the person, they should offer to help them find someone else to talk to.

Essential	Important	Don't know/Depends	Unimportant	Should not be included
○	○	○	○	○

34. If the first aider feels uncomfortable interacting with the person due to differences in age group or gender, they should seek assistance from someone of the same age group or gender as the person.

Essential	Important	Don't know/Depends	Unimportant	Should not be included
○	○	○	○	○

35. If the first aider feels uncomfortable interacting with the person due to differences in cultural background, they should seek the assistance of someone of the same cultural background as the person.

Essential	Important	Don't know/Depends	Unimportant	Should not be included
○	○	○	○	○

Section 1. Identification of suicide risk continued (4)

Reacting to expressions of suicidal thoughts

The first aider should:

	Essential	Important	Don't know/Depends	Unimportant	Should not be included
36. Know that it is common to feel panic or shock when someone discloses thoughts of suicide.	◯	◯	◯	◯	◯
37. Appear calm and confident in the face of the suicide crisis, as this may have a reassuring effect for the suicidal person.	◯	◯	◯	◯	◯
38. React to expressions of suicidal thoughts with calmness and empathy.	◯	◯	◯	◯	◯
39. Avoid expressing negative reactions to suicidal thoughts, e.g. judgement, shock, panic, anger.	◯	◯	◯	◯	◯
40. Respect the suicidal person and not try to take charge of the situation.	◯	◯	◯	◯	◯
41. Allow the suicidal person to discuss their feelings. A suicidal person may feel relief at being able to do so.	◯	◯	◯	◯	◯
42. Know that if the distressed person says they are not suicidal, they probably are not.	◯	◯	◯	◯	◯

43. If the person is at a point of despair, the first aider needs to take control and be directive in ensuring their safety.

Essential	Important	Don't know/Depends	Unimportant	Should not be included
◯	◯	◯	◯	◯

44. If the first aider clearly states that thoughts of suicide may be associated with a treatable disorder, this may instil a sense of hope for the suicidal person.

Essential	Important	Don't know/Depends	Unimportant	Should not be included
◯	◯	◯	◯	◯

If the person is experiencing an episode of psychosis

45. If the person is psychotic, the first aider may not be able to believe them if they say they are not suicidal.

Essential	Important	Don't know/Depends	Unimportant	Should not be included
◯	◯	◯	◯	◯

46. If the suicidal person says they are hearing voices, the first aider should ask if the voices are telling them to kill themselves.

Essential	Important	Don't know/Depends	Unimportant	Should not be included
◯	◯	◯	◯	◯

If the person is under the influence of drugs & alcohol

47. If the person is using drugs or alcohol, the first aider may not be able to believe them if they say they are not suicidal.

Essential	Important	Don't know/Depends	Unimportant	Should not be included
○	○	○	○	○

Do you have any comments on these statements? Is there anything you would like to add to this section? Please write your ideas in the box provided

Section 2. Assessing seriousness of the suicide risk

This section contains statements about accessing the seriousness of the person's risk of suicide.

Please rate how important (from *essential* to *should not be included*) you think it is that each statement be included in the final guidelines.

Assessing the urgency of suicide risk

48. The first aider should take all thoughts of suicide seriously. The lack of a plan for suicide is not sufficient to ensure safety.

Essential	Important	Don't know/Depends	Unimportant	Should not be included
○	○	○	○	○

49. The first aider should determine the urgency of taking action based on recognition of suicide warning signs.

Essential	Important	Don't know/Depends	Unimportant	Should not be included
○	○	○	○	○

50. The first aider should take expressions of suicidal thoughts seriously and act on these, not dismissing them as 'attention seeking' or a 'cry for help'.

Essential	Important	Don't know/Depends	Unimportant	Should not be included
○	○	○	○	○

51. The first aider should ask the suicidal person if they are really serious or just looking for attention.

Essential	Important	Don't know/Depends	Unimportant	Should not be included
◯	◯	◯	◯	◯

52. The first aider should establish whether the person has definite plans and intentions to take their life as opposed to vague suicidal notions such as "what's the point?" or "I can't be bothered going on".

Essential	Important	Don't know/Depends	Unimportant	Should not be included
◯	◯	◯	◯	◯

53. If the suicidal person says that the situation is not serious or that they can handle it on their own, the first aider should respect this.

Essential	Important	Don't know/Depends	Unimportant	Should not be included
◯	◯	◯	◯	◯

54. The first aider should not let the suicidal person convince them that it is not serious or that they can handle it on their own.

Essential	Important	Don't know/Depends	Unimportant	Should not be included
◯	◯	◯	◯	◯

Finding out about a suicide plan

The first aider should:

	Essential	Important	Don't know/Depends	Unimportant	Should not be included
55. Ask the suicidal person if they have a plan for suicide.	◯	◯	◯	◯	◯
56. Ask the suicidal person how they intend to suicide i.e. ask them direct questions about how, when and where they intend to suicide.	◯	◯	◯	◯	◯
57. Ask the suicidal person if they have decided when they will carry out their plan.	◯	◯	◯	◯	◯
58. Find out if the suicidal person has already taken steps to secure the means to end their life.	◯	◯	◯	◯	◯
59. Be aware that those at the highest risk for acting on thoughts of suicide in the near future have a specific suicide plan, the means to carry out the plan, a time set for doing it, and an intention to do it.	◯	◯	◯	◯	◯

Asking about other factors that contribute to risk

60. The first aider should be able to recognise the person's level of suicide risk by the number and nature of warning signs.

Essential	Important	Don't know/Depends	Unimportant	Should not be included
○	○	○	○	○

The first aider should ask the suicidal person:

	Essential	Important	Don't know/Depends	Unimportant	Should not be included
61. If they have been using drugs or alcohol.	○	○	○	○	○
62. Ask if the suicidal person has received treatment for mental health problems or is taking any medication.	○	○	○	○	○
63. If they have received mental health treatment in the past.	○	○	○	○	○
64. If they have ever known anyone who has died by suicide.	○	○	○	○	○
65. About any family history of mental health problems or suicide.	○	○	○	○	○
66. If they have ever made a suicide plan in the past.	○	○	○	○	○
67. If they have ever made a suicide attempt in the past.	○	○	○	○	○
68. How they are feeling right now.	○	○	○	○	○
69. If they have told anyone about how they are feeling.	○	○	○	○	○
70. How things are at home and work/school.	○	○	○	○	○
71. If there have been changes in their employment, social life, or family.	○	○	○	○	○
72. About current supports to help the person at risk.	○	○	○	○	○

73. The first aider should avoid asking the suicidal person if they have a 'mental illness', instead asking if they are receiving help for any emotional or mental health problems.

Essential	Important	Don't know/Depends	Unimportant	Should not be included
○	○	○	○	○

Do you have any comments on these statements? Is there anything you would like to add to this section? Please write your ideas in the box provided

Section 3. Initial assistance

This section contains statements about the assistance to be first provided to the person.

Please rate how important (from *essential* to *should not be included*) you think it is that each statement be included in the final guidelines.

74. The first aider should only try to assist a suicidal person with whom they have a close relationship.

Essential	Important	Don't know/Depends	Unimportant	Should not be included
◯	◯	◯	◯	◯

75. The first aider should not put themselves in any danger while offering support to the suicidal person.

Essential	Important	Don't know/Depends	Unimportant	Should not be included
◯	◯	◯	◯	◯

76. The first aider should not leave someone who is feeling suicidal on their own.

Essential	Important	Don't know/Depends	Unimportant	Should not be included
◯	◯	◯	◯	◯

77. The first aider does not need to be with the suicidal person all the time, but should check on them regularly.

Essential	Important	Don't know/Depends	Unimportant	Should not be included
◯	◯	◯	◯	◯

78. The first aider should work collaboratively with the suicidal person to ensure their safety, rather than acting alone to prevent suicide.

Essential	Important	Don't know/Depends	Unimportant	Should not be included
◯	◯	◯	◯	◯

79. If the first aider suspects there is an immediate risk of the person acting on suicidal thoughts, they should act quickly even if they are unsure.

Essential	Important	Don't know/Depends	Unimportant	Should not be included
◯	◯	◯	◯	◯

80. The first aider should be prepared for the suicidal person to possibly express anger and feel betrayed by their attempt to prevent their suicide or help them get professional help.

Essential	Important	Don't know/Depends	Unimportant	Should not be included
○	○	○	○	○

81. If the person is suicidal, the first aider should:

	Essential	Important	Don't know/Depends	Unimportant	Should not be included
Discuss with the suicidal person what actions they should take to get help.	○	○	○	○	○
Encourage the suicidal person to get appropriate professional help as soon as possible (i.e. See a mental health professional or someone at a mental health service).	○	○	○	○	○
Seek the permission of the suicidal person to contact their regular doctor or mental health professional about their concerns.	○	○	○	○	○
Call a mental health centre or crisis telephone line and ask for advice on the situation.	○	○	○	○	○
Call a doctor, psychiatrist or other professional right away for the suicidal person.	○	○	○	○	○
Get the suicidal person to phone an emergency number (i.e. Emergency services, a suicide helpline, emergency mental health services).	○	○	○	○	○
Phone an emergency number **without letting the suicidal person know**.	○	○	○	○	○
Take the suicidal person to a hospital emergency department.	○	○	○	○	○

Section 3. Initial assistance (2)

82. If the suicidal person can't commit to stay safe, the first aider should:

	Essential	Important	Don't know/Depends	Unimportant	Should not be included
Discuss with the suicidal person what actions they should take to get help.	○	○	○	○	○
Encourage the suicidal person to get appropriate professional help as soon as possible (i.e. See a mental health professional or someone at a mental health service).	○	○	○	○	○
Seek the permission of the suicidal person to contact their regular doctor or mental health professional about their concerns.	○	○	○	○	○
Call a mental health centre or crisis telephone line and ask for advice on the situation.	○	○	○	○	○
Call a doctor, psychiatrist or other professional right away for the suicidal person.	○	○	○	○	○
Get the suicidal person to phone an emergency number (i.e. Emergency services, a suicide helpline, emergency mental health services).	○	○	○	○	○
Phone an emergency number **without letting the suicidal person know**.	○	○	○	○	○
Take the suicidal person to a hospital emergency department.	○	○	○	○	○

83. If the suicidal person has a specific plan, the first aider should:

	Essential	Important	Don't know/Depends	Unimportant	Should not be included
Discuss with the suicidal person what actions they should take to get help.	○	○	○	○	○
Encourage the suicidal person to get appropriate professional help as soon as possible (i.e. See a mental health professional or someone at a mental health service).	○	○	○	○	○
Seek the permission of the suicidal person to contact their regular doctor or mental health professional about their concerns.	○	○	○	○	○
Call a mental health centre or crisis telephone line and ask for advice on the situation.	○	○	○	○	○
Call a doctor, psychiatrist or other professional right away for the suicidal person.	○	○	○	○	○
Get the suicidal person to phone an emergency number (i.e. Emergency services, a suicide helpline, emergency mental health services).	○	○	○	○	○
Phone an emergency number **without letting the suicidal person know**.	○	○	○	○	○
Take the suicidal person to a hospital emergency department.	○	○	○	○	○

84. If the suicidal person has the means to carry out their suicide plan, the first aider should:

	Essential	Important	Don't know/Depends	Unimportant	Should not be included
Discuss with the suicidal person what actions they should take to get help.	○	○	○	○	○
Encourage the suicidal person to get appropriate professional help as soon as possible (i.e. See a mental health professional or someone at a mental health service).	○	○	○	○	○
Seek the permission of the suicidal person to contact their regular doctor or mental health professional about their concerns.	○	○	○	○	○
Call a mental health centre or crisis telephone line and ask for advice on the situation.	○	○	○	○	○
Call a doctor, psychiatrist or other professional right away for the suicidal person.	○	○	○	○	○
Get the suicidal person to phone an emergency number (i.e. Emergency services, a suicide helpline, emergency mental health services).	○	○	○	○	○
Phone an emergency number **without letting the suicidal person know**.	○	○	○	○	○
Take the suicidal person to a hospital emergency department.	○	○	○	○	○

85. If the suicidal person does not agree to give the first aider the things they intend using to kill themselves, the first aider should:

	Essential	Important	Don't know/Depends	Unimportant	Should not be included
Discuss with the suicidal person what actions they should take to get help.	○	○	○	○	○
Encourage the suicidal person to get appropriate professional help as soon as possible (i.e. See a mental health professional or someone at a mental health service).	○	○	○	○	○
Seek the permission of the suicidal person to contact their regular doctor or mental health professional about their concerns.	○	○	○	○	○
Call a mental health centre or crisis telephone line and ask for advice on the situation.	○	○	○	○	○
Call a doctor, psychiatrist or other professional right away for the suicidal person.	○	○	○	○	○
Get the suicidal person to phone an emergency number (i.e. Emergency services, a suicide helpline, emergency mental health services).	○	○	○	○	○
Phone an emergency number **without letting the suicidal person know**.	○	○	○	○	○
Take the suicidal person to a hospital emergency department.	○	○	○	○	○

86. If the suicidal person has attempted suicide in the past, the first aider should:

	Essential	Important	Don't know/Depends	Unimportant	Should not be included
Discuss with the suicidal person what actions they should take to get help.	○	○	○	○	○
Encourage the suicidal person to get appropriate professional help as soon as possible (i.e. See a mental health professional or someone at a mental health service).	○	○	○	○	○
Seek the permission of the suicidal person to contact their regular doctor or mental health professional about their concerns.	○	○	○	○	○
Call a mental health centre or crisis telephone line and ask for advice on the situation.	○	○	○	○	○
Call a doctor, psychiatrist or other professional right away for the suicidal person.	○	○	○	○	○
Get the suicidal person to phone an emergency number (i.e. Emergency services, a suicide helpline, emergency mental health services).	○	○	○	○	○
Phone an emergency number **without letting the suicidal person know**.	○	○	○	○	○
Take the suicidal person to a hospital emergency department.	○	○	○	○	○

Section 3. Initial assistance (3)

87. If the suicidal person is known to have a diagnosis of a mental illness, the first aider should:

	Essential	Important	Don't know/Depends	Unimportant	Should not be included
Discuss with the suicidal person what actions they should take to get help.	○	○	○	○	○
Encourage the suicidal person to get appropriate professional help as soon as possible (i.e. See a mental health professional or someone at a mental health service).	○	○	○	○	○
Seek the permission of the suicidal person to contact their regular doctor or mental health professional about their concerns.	○	○	○	○	○
Call a mental health centre or crisis telephone line and ask for advice on the situation.	○	○	○	○	○
Call a doctor, psychiatrist or other professional right away for the suicidal person.	○	○	○	○	○
Get the suicidal person to phone an emergency number (i.e. Emergency services, a suicide helpline, emergency mental health services).	○	○	○	○	○
Phone an emergency number **without letting the suicidal person know**.	○	○	○	○	○
Take the suicidal person to a hospital emergency department.	○	○	○	○	○

88. If the suicidal person is psychotic, the first aider should:

	Essential	Important	Don't know/Depends	Unimportant	Should not be included
Discuss with the suicidal person what actions they should take to get help.	◯	◯	◯	◯	◯
Encourage the suicidal person to get appropriate professional help as soon as possible (i.e. See a mental health professional or someone at a mental health service).	◯	◯	◯	◯	◯
Seek the permission of the suicidal person to contact their regular doctor or mental health professional about their concerns.	◯	◯	◯	◯	◯
Call a mental health centre or crisis telephone line and ask for advice on the situation.	◯	◯	◯	◯	◯
Call a doctor, psychiatrist or other professional right away for the suicidal person.	◯	◯	◯	◯	◯
Get the suicidal person to phone an emergency number (i.e. Emergency services, a suicide helpline, emergency mental health services).	◯	◯	◯	◯	◯
Phone an emergency number **without letting the suicidal person know**.	◯	◯	◯	◯	◯
Take the suicidal person to a hospital emergency department.	◯	◯	◯	◯	◯

89. If the suicidal person refuses professional help, the first aider should:

	Essential	Important	Don't know/Depends	Unimportant	Should not be included
Discuss with the suicidal person what actions they should take to get help.	◯	◯	◯	◯	◯
Encourage the suicidal person to get appropriate professional help as soon as possible (i.e. See a mental health professional or someone at a mental health service).	◯	◯	◯	◯	◯
Seek the permission of the suicidal person to contact their regular doctor or mental health professional about their concerns.	◯	◯	◯	◯	◯
Call a mental health centre or crisis telephone line and ask for advice on the situation.	◯	◯	◯	◯	◯
Call a doctor, psychiatrist or other professional right away for the suicidal person.	◯	◯	◯	◯	◯
Get the suicidal person to phone an emergency number (i.e. Emergency services, a suicide helpline, emergency mental health services).	◯	◯	◯	◯	◯
Phone an emergency number **without letting the suicidal person know**.	◯	◯	◯	◯	◯
Take the suicidal person to a hospital emergency department.	◯	◯	◯	◯	◯

90. If the first aider needs to contact a health professional about the suicidal person, they should preferably contact a professional the person already knows and trusts.

Essential	Important	Don't know/Depends	Unimportant	Should not be included
◯	◯	◯	◯	◯

91. If the first aider has to call the police, they should inform them that the person is suicidal in order to help them respond appropriately.

Essential	Important	Don't know/Depends	Unimportant	Should not be included
◯	◯	◯	◯	◯

92. If the suicidal person is reluctant to seek help, the first aider should:

	Essential	Important	Don't know/Depends	Unimportant	Should not be included
Keep encouraging them to see a mental health professional.	◯	◯	◯	◯	◯
Seek an agreement with them that they will contact a specific person within a specified timeframe.	◯	◯	◯	◯	◯
Contact a suicide prevention hotline for guidance on how to help them.	◯	◯	◯	◯	◯
Talk to a health professional for advice on the situation.	◯	◯	◯	◯	◯
Contact emergency services on their behalf.	◯	◯	◯	◯	◯
Make sure someone who is close to the suicidal person is aware of the situation (i.e. close friend or family member).	◯	◯	◯	◯	◯

93. If the suicidal person doesn't want to talk to someone face-to-face, the first aider should encourage them to contact a suicide helpline.

Essential	Important	Don't know/Depends	Unimportant	Should not be included
◯	◯	◯	◯	◯

94. The first aider should find out information on the resources and services available for a person who is considering suicide.

Essential	Important	Don't know/Depends	Unimportant	Should not be included
◯	◯	◯	◯	◯

95. The first aider should provide the suicidal person with information and resources about where they can seek help.

Essential	Important	Don't know/Depends	Unimportant	Should not be included
○	○	○	○	○

96. If the suicidal person is willing to seek professional help for their suicidal thoughts, the first aider should help them plan what they will say.

Essential	Important	Don't know/Depends	Unimportant	Should not be included
○	○	○	○	○

Section 3. Initial assistance (4)

97. If the suicidal person has a weapon, the first aider should:

	Essential	Important	Don't know/Depends	Unimportant	Should not be included
Try to take it away from them.	○	○	○	○	○
Ask them to hand over the weapon.	○	○	○	○	○
Not try to take it away from them.	○	○	○	○	○
Remove themselves from the situation.	○	○	○	○	○
Contact the police.	○	○	○	○	○

98. If possible, the first aider should measure out prescription medication so that the suicidal person only has a certain amount available (e.g. couple of days' worth).

Essential	Important	Don't know/Depends	Unimportant	Should not be included
○	○	○	○	○

99. If the conversation about suicide is taking place on the phone, the first aider should encourage the suicidal person to remove potentially harmful items from their sight.

Essential	Important	Don't know/Depends	Unimportant	Should not be included
○	○	○	○	○

100. If the conversation about suicide is taking place on the phone, the first aider should contact emergency services so the person is not alone.

Essential	Important	Don't know/Depends	Unimportant	Should not be included
○	○	○	○	○

101. The first aider must keep in mind that they may not be successful in preventing suicide.

Essential	Important	Don't know/Depends	Unimportant	Should not be included
○	○	○	○	○

Do you have any comments on these statements? Is there anything you would like to add to this section? Please write your ideas in the box provided

Section 4. Talking with a suicidal person

This section contains statements about talking to the suicidal person about their suicidal thoughts.

Please rate how important (from *essential* to *should not be included*) you think it is that each statement be included in the final guidelines.

Letting them know you care

The first aider should:

	Essential	Important	Don't know/Depends	Unimportant	Should not be included
102. Tell the suicidal person they care and want to help.	◯	◯	◯	◯	◯
103. Tell the suicidal person that they do not want them to die.	◯	◯	◯	◯	◯
104. Remind the suicidal person that they are loved and would be missed.	◯	◯	◯	◯	◯
105. Reassure the suicidal person that they want to hear whatever the person has to say.	◯	◯	◯	◯	◯
106. Give the suicidal person their undivided attention.	◯	◯	◯	◯	◯
107. Be patient and calm while the suicidal person is talking about their feelings.	◯	◯	◯	◯	◯
108. Be conscious of their body language, ensuring it doesn't communicate a lack of interest or negative attitude.	◯	◯	◯	◯	◯
109. Keep in mind that asking too many questions can provoke anxiety in the suicidal person.	◯	◯	◯	◯	◯
110. Show they are listening by summarising what the suicidal person is saying.	◯	◯	◯	◯	◯
111. Clarify important points with the person to make sure they fully understand.	◯	◯	◯	◯	◯
112. Ask what the suicidal person is thinking and feeling.	◯	◯	◯	◯	◯
113. Be supportive and understanding of the suicidal person.	◯	◯	◯	◯	◯
114. Express empathy for the suicidal person.	◯	◯	◯	◯	◯
115. Listen to the suicidal person without expressing judgment.	◯	◯	◯	◯	◯

Actively listening

The first aider should:

	Essential	Important	Don't know/Depends	Unimportant	Should not be included
116. Encourage the suicidal person to do most of the talking.	◯	◯	◯	◯	◯
117. Let the suicidal person know that it's okay to talk about things that might be painful.	◯	◯	◯	◯	◯
118. Ask open questions to find out more about the suicidal thoughts and feelings and the problems behind these.	◯	◯	◯	◯	◯
119. Share their thoughts with the suicidal person without expressing judgement.	◯	◯	◯	◯	◯
119. Focus on the things that will keep the suicidal person safe for now rather than the things that put the person at risk.	◯	◯	◯	◯	◯
120. Avoid discussion of any mental health problems experienced by the suicidal person, focusing instead on the reasons behind the suicide crisis.	◯	◯	◯	◯	◯

Reacting to suicidal thoughts

121. Suicidal thoughts are often a plea for help and a desperate attempt to escape from problems and distressing feelings. The first aider should therefore allow the suicidal person to talk about those thoughts and feelings.

Essential	Important	Don't know/Depends	Unimportant	Should not be included
◯	◯	◯	◯	◯

122. The first aider needs to allow the suicidal person to talk about their reasons for wanting to die.

Essential	Important	Don't know/Depends	Unimportant	Should not be included
◯	◯	◯	◯	◯

The first aider should:

	Essential	Important	Don't know/Depends	Unimportant	Should not be included
123. Encourage the suicidal person to discuss their reasons for dying and their reasons for living, validate that they are considering both options and emphasise that living is an option for them.	◯	◯	◯	◯	◯
124. Ask about issues that affect the immediate safety of the person who is suicidal.	◯	◯	◯	◯	◯
125. Accept what the suicidal person is saying without agreeing or disagreeing with their behaviour or point of view.	◯	◯	◯	◯	◯
126. Validate the suicidal person's thoughts and feelings and acknowledge that these may be hard to talk about.	◯	◯	◯	◯	◯
127. Allow the suicidal person to express their feelings (.e.g. allow them to cry, express anger or scream).	◯	◯	◯	◯	◯
128. Say whatever they feel they need to help the suicidal person decide against suicide, including the use of guilt and threats, e.g. telling them they will go to hell or they will ruin the lives of others if they die by suicide.	◯	◯	◯	◯	◯

Offering reassurance

The first aider should:

	Essential	Important	Don't know/Depends	Unimportant	Should not be included
129. Reassure the suicidal person that it's okay to feel the way they do.	○	○	○	○	○
130. Tell the person to cheer up, and promise that everything will be ok.	○	○	○	○	○
131. Offer hope and reassurance that their feelings are temporary, that help is available and things will get better.	○	○	○	○	○
132. Assure the suicidal person that the feelings they are experiencing are probably caused by a mental illness that can be treated.	○	○	○	○	○
133. Reassure the suicidal person that they understand how badly they feel.	○	○	○	○	○
134. Reassure the suicidal person that they are there for them and want to help.	○	○	○	○	○
135. Reassure the suicidal person that thoughts of suicide are common, and that many people have them at some stage in their lives.	○	○	○	○	○
136. Reassure the suicidal person by letting them know that we all go through tough times and need support and that reaching out for help is the first step to feeling better.	○	○	○	○	○
137. Remind the suicidal person that suicidal thoughts need not be acted on.	○	○	○	○	○
138. Reassure the suicidal person that there are solutions to problems or ways other than suicide for coping.	○	○	○	○	○

Highlighting protective factors

139. The fact that the suicidal person is still alive, and talking to the first aider about their feelings, means that they are not quite sure about suicide. The first aider should point this out as a positive thing.

Essential	Important	Don't know/Depends	Unimportant	Should not be included
○	○	○	○	○

The first aider should:

	Essential	Important	Don't know/Depends	Unimportant	Should not be included
140. Thank the suicidal person for sharing their feelings with them and acknowledging the courage this takes.	○	○	○	○	○
141. Discuss the 'good things' in a person's life, their hopes for the future, and other reasons to live.	○	○	○	○	○
142. Find out what has supported the suicidal person in the past and whether these supports are still available.	○	○	○	○	○
143. Encourage the suicidal person to think about their personal strengths and the positive things in their life.	○	○	○	○	○
144. Remind the person of the good qualities they have.	○	○	○	○	○

Section 4. Talking with a suicidal person (2)

Helping with problem-solving

145. The first aider should inquire about the problems the suicidal person is facing and how they can help.

Essential	Important	Don't know/Depends	Unimportant	Should not be included
○	○	○	○	○

146. The first aider should acknowledge suicide as an option, but indicate there are other alternatives.

Essential	Important	Don't know/Depends	Unimportant	Should not be included
○	○	○	○	○

147. It is important that the first aider dispute the idea that suicide is the best or most viable solution.

Essential	Important	Don't know/Depends	Unimportant	Should not be included
○	○	○	○	○

148. In order to reduce suicide risk, it is important for the first aider to try to solve the suicidal person's problems.

Essential	Important	Don't know/Depends	Unimportant	Should not be included
○	○	○	○	○

149. By discussing specific problems, the first aider can help the person work out ways of dealing with the difficulties that seem insurmountable.

Essential	Important	Don't know/Depends	Unimportant	Should not be included
○	○	○	○	○

150. The first aider should assist the suicidal person with problem-solving, by asking about the problem, identifying available resources, suggesting solutions and a plan of action.

Essential	Important	Don't know/Depends	Unimportant	Should not be included
○	○	○	○	○

151. The first aider should help the suicidal person put their problems into perspective by reminding them that other people have much worse problems and still choose to live.

Essential	Important	Don't know/Depends	Unimportant	Should not be included
○	○	○	○	○

152. The first aider should not try to provide a solution to the suicidal person's problems.

Essential	Important	Don't know/Depends	Unimportant	Should not be included
○	○	○	○	○

Considering the consequences of suicide

The first aider should:

	Essential	Important	Don't know/Depends	Unimportant	Should not be included
153. Encourage the suicidal person to consider the consequences of suiciding, especially the effect it may have on the people they care about.	○	○	○	○	○
154. Remind the suicidal person that suicide is a permanent solution to a temporary problem.	○	○	○	○	○
155. Accept the suicidal feelings for what they are and discuss suicide as a possibility rather than an unthinkable act.	○	○	○	○	○

Physical contact

156. If the first aider has a close relationship with the suicidal person, they should show they care about them by hugging them or holding their hand.

Essential	Important	Don't know/Depends	Unimportant	Should not be included
○	○	○	○	○

157. The first aider should not touch (e.g. hug or hold hands with) the suicidal person unless they have a close personal relationship.

Essential	Important	Don't know/Depends	Unimportant	Should not be included
○	○	○	○	○

158. The first aider should not touch (e.g. hug or hold hands with) the suicidal person without their permission.

Essential	Important	Don't know/Depends	Unimportant	Should not be included
○	○	○	○	○

Offering support

159. The first aider should ask the suicidal person how they would like to be supported and if there is anything they can do to help.

Essential	Important	Don't know/Depends	Unimportant	Should not be included
○	○	○	○	○

160. The first aider should not try to take on the suicidal person's responsibilities.

Essential	Important	Don't know/Depends	Unimportant	Should not be included
○	○	○	○	○

161. The first aider should suggest things to distract the suicidal person from their suicidal thoughts, especially things which are relatively easy to do and which will encourage a sense of control and achievement.

Essential	Important	Don't know/Depends	Unimportant	Should not be included
○	○	○	○	○

162. If the first aider is having trouble communicating with the suicidal person, they should ask simple questions, repeating these if necessary.

Essential	Important	Don't know/Depends	Unimportant	Should not be included
○	○	○	○	○

Offer practical help

163. The first aider should offer to help the suicidal person with practical tasks. This can give the person a chance to spend some time dealing with their situation or give them a chance for some rest.

Essential	Important	Don't know/Depends	Unimportant	Should not be included
○	○	○	○	○

Section 4. Talking with a suicidal person (3)

What to avoid when talking with a suicidal person

164. The first aider should NOT:

	Essential	Important	Don't know/Depends	Unimportant	Should not be included
Argue or debate with the person about their thoughts of suicide.	○	○	○	○	○
Discuss with the person whether suicide is right or wrong.	○	○	○	○	○
Minimise the suicidal person's problems.	○	○	○	○	○
Give glib 'reassurance' such as 'don't worry', 'cheer up', 'you have everything going for you' or 'everything will be alright'.	○	○	○	○	○
Interrupt with stories of their own.	○	○	○	○	○
'Call their bluff', dare or tell the suicidal person to 'just do it'.	○	○	○	○	○
Attempt to give the suicidal person a diagnosis of a mental health problem.	○	○	○	○	○
Use guilt or threats to prevent suicide (e.g. do not tell the person they will go to hell or ruin other people's lives if they die by suicide).	○	○	○	○	○
Let the fear of saying the wrong words or of not saying the perfect words keep them from encouraging the suicidal person to talk.	○	○	○	○	○
Take any hurtful actions or words of the suicidal person personally.	○	○	○	○	○

165. The first aider should avoid giving advice.

Essential	Important	Don't know/Depends	Unimportant	Should not be included
○	○	○	○	○

166. The first aider should avoid asking the suicidal person why they are having suicidal thoughts.

Essential	Important	Don't know/Depends	Unimportant	Should not be included
○	○	○	○	○

167. If the suicidal person is thinking about taking an overdose, the first aider should tell the person that overdosing can lead to messy, painful and long-drawn-out consequences, such as slow poisoning.

Essential	Important	Don't know/Depends	Unimportant	Should not be included
○	○	○	○	○

Do you have any comments on these statements? Is there anything you would like to add to this section? Please write your ideas in the box provided

Section 5. No-suicide contracts & safety planning

The statements in this section are about the development of no-suicide contracts and planning to keep the suicidal person safe.

Please keep in mind that a **Safety plan** is an agreement between the suicidal person and the first aider that involves actions to keep the suicidal person safe.

Please rate how important (from *essential* to *should not be included*) you think it is that each statement be included in the final guidelines.

Developing a safety plan

168. The first aider should develop a safety plan with the suicidal person.

Essential	Important	Don't know/Depends	Unimportant	Should not be included
○	○	○	○	○

169. The first aider should engage the suicidal person to the fullest extent possible in decisions about a safety plan.

Essential	Important	Don't know/Depends	Unimportant	Should not be included
○	○	○	○	○

170. The safety plan should be written down and signed by both the first aider and the suicidal person.

Essential	Important	Don't know/Depends	Unimportant	Should not be included
○	○	○	○	○

171. If the suicidal person doesn't want to write the safety plan down, it is fine to make a verbal agreement.

Essential	Important	Don't know/Depends	Unimportant	Should not be included
○	○	○	○	○

172. Verbal agreements should be repeated out loud by the suicidal person back to the first aider to ensure that both parties agree on the terms.

Essential	Important	Don't know/Depends	Unimportant	Should not be included
○	○	○	○	○

173. The first aider should get a verbal commitment from the suicidal person that they will not act upon thoughts of suicide until they have met with a professional.

Essential	Important	Don't know/Depends	Unimportant	Should not be included
○	○	○	○	○

What the plan should include

The safety plan should:

	Essential	Important	Don't know/Depends	Unimportant	Should not be included
174. Be clear, outlining what will be done, who will be doing it, and when it will be carried out.	○	○	○	○	○
175. Focus more on what the suicidal person should do rather than what they should not do.	○	○	○	○	○
176. Include an agreement that the suicidal person does not attempt suicide.	○	○	○	○	○
177. Include an agreement that the suicidal person does not use any alcohol or other drugs.	○	○	○	○	○
178. Be kept somewhere accessible to the suicidal person.	○	○	○	○	○

179. The first aider should not assume that a safety plan is adequate to keep the suicidal person safe.

Essential	Important	Don't know/Depends	Unimportant	Should not be included
○	○	○	○	○

Section 5. No-suicide contracts (2)

Who to contact

180. Safety plans should include 24-hour safety contacts in case the suicidal person feels unable to continue with the agreement not to attempt suicide (such as a suicide helpline, professional helper or family member).

Essential	Important	Don't know/Depends	Unimportant	Should not be included
○	○	○	○	○

181. The safety plan should include contact numbers for the suicidal person's doctor or mental health care professional, a suicide helpline or 24-hour crisis line, as well as friends and family members who will help in an emergency.

Essential	Important	Don't know/Depends	Unimportant	Should not be included
○	○	○	○	○

182. The first aider should ask the suicidal person to keep a list of safety contacts with them and agree to call someone when they are feeling suicidal.

Essential	Important	Don't know/Depends	Unimportant	Should not be included
○	○	○	○	○

Timeframe of the safety plan

The safety plan should:

	Essential	Important	Don't know/Depends	Unimportant	Should not be included
183. Include an indication of when they will end, for example, the time the suicidal person will next speak to the first aider.	○	○	○	○	○
184. Be for a length of time which will be easy for the suicidal person to cope with, so that they can feel able to fulfil the agreement and have a sense of achievement.	○	○	○	○	○
185. Be for a length of time that will present a challenge for the suicidal person, so that they can see that they can manage alone.	○	○	○	○	○

186. The first aider should work with the suicidal person to create plans to ensure their safety for the next 24, 48 and 72 hours.

Essential	Important	Don't know/Depends	Unimportant	Should not be included
○	○	○	○	○

When to make a safety plan

187. If the suicidal person won't make a safety plan, it is not safe to leave them alone for any period of time. The first aider should make sure someone stays close by the person (in the same room, in visual contact) and get outside help immediately.

Essential	Important	Don't know/Depends	Unimportant	Should not be included
◯	◯	◯	◯	◯

188. If the suicidal person won't make a safety plan, the first aider should get professional help immediately.

Essential	Important	Don't know/Depends	Unimportant	Should not be included
◯	◯	◯	◯	◯

189. The first aider shouldn't use a safety plan with a suicidal person they don't know well.

Essential	Important	Don't know/Depends	Unimportant	Should not be included
◯	◯	◯	◯	◯

190. The first aider should only make a safety plan with someone they know well.

Essential	Important	Don't know/Depends	Unimportant	Should not be included
◯	◯	◯	◯	◯

191. The first aider shouldn't use a safety plan with a suicidal person who is severely depressed.

Essential	Important	Don't know/Depends	Unimportant	Should not be included
◯	◯	◯	◯	◯

192. The first aider shouldn't use a safety plan with a suicidal person who is using drugs or alcohol.

Essential	Important	Don't know/Depends	Unimportant	Should not be included
◯	◯	◯	◯	◯

193. The first aider shouldn't use a safety plan with a suicidal person who is psychotic.

Essential	Important	Don't know/Depends	Unimportant	Should not be included
◯	◯	◯	◯	◯

Do you have any comments on these statements? Is there anything you would like to add to this section? Please write your ideas in the box provided

Section 6. Ensuring safety

The statements in this section contain actions the first aider can take to help ensure the safety of the suicidal person.

Please rate how important (from *essential* to *should not be included*) you think it is that each statement be included in the final guidelines.

194. The first aider should make sure any potentially harmful items are not available to the suicidal person by removing access to these items.

Essential	Important	Don't know/Depends	Unimportant	Should not be included
○	○	○	○	○

195. The first aider should try to remove the means of suicide available to the suicidal person if it is safe to do so.

Essential	Important	Don't know/Depends	Unimportant	Should not be included
○	○	○	○	○

196. The first aider should ask the suicidal person to give them the things they intend using to kill themselves.

Essential	Important	Don't know/Depends	Unimportant	Should not be included
○	○	○	○	○

197. If the suicidal person agrees to give the first aider the things they intend using to kill themselves, the first aider should dispose of them right away (i.e. flush pills down the toilet, hand gun to the police, throw away razors or knives).

Essential	Important	Don't know/Depends	Unimportant	Should not be included
○	○	○	○	○

198. If the suicidal person agrees to hand over the means of suicide, on the condition that they can have them back if they want them, the first aider should argue the point with them for as long as it takes.

Essential	Important	Don't know/Depends	Unimportant	Should not be included
○	○	○	○	○

199. If the suicidal person agrees to hand over the means of suicide, on the condition that they can have them back if they want them, the first aider should agree to this.

Essential	Important	Don't know/Depends	Unimportant	Should not be included
○	○	○	○	○

200. If the first aider can't get the suicidal person to agree to hand over the means of suicide (for example, pills, gun, razor), emergency services must be contacted immediately.

Essential	Important	Don't know/Depends	Unimportant	Should not be included
○	○	○	○	○

201. If the first aider can't get the suicidal person to agree to hand over the means of suicide (for example, pills, gun, razor), they should try to take these things secretly.

Essential	Important	Don't know/Depends	Unimportant	Should not be included
○	○	○	○	○

Do you have any comments on these statements? Is there anything you would like to add to this section? Please write your ideas in the box provided

Section 7. Passing time during a crisis

The statements in this section are about what the first aider should do with the suicidal person during the suicide crisis period.

Please rate how important (from *essential* to *should not be included*) you think it is that each statement be included in the final guidelines.

The first aider should:

	Essential	Important	Don't know/Depends	Unimportant	Should not be included
202. Ask the suicidal person to postpone the decision to suicide.	◯	◯	◯	◯	◯
203. Develop a list with the suicidal person of other things they can do to distract themselves.	◯	◯	◯	◯	◯
204. Do something pleasant for the suicidal person. For example, cooking a favourite meal, renting a movie or listening to music with them.	◯	◯	◯	◯	◯
205. Encourage the suicidal person to undertake some relaxing activities, such as taking a hot bath, going for a long walk or reading something enjoyable.	◯	◯	◯	◯	◯
206. Encourage the suicidal person to do something active like going for a swim or a jog.	◯	◯	◯	◯	◯
207. Offer to join the person in some activity they normally enjoy.	◯	◯	◯	◯	◯
208. Encourage the suicidal person to take some sleeping pills, as they should be feeling better by the time they wake up.	◯	◯	◯	◯	◯
209. Encourage the suicidal person to drink a few glasses of alcohol, to make the time pass more quickly.	◯	◯	◯	◯	◯

210. The first aider should not take the suicidal person to parties or places where people are having fun, as this could make them more depressed.

Essential	Important	Don't know/Depends	Unimportant	Should not be included
◯	◯	◯	◯	◯

211. During the suicidal crisis, the suicidal person and the first aider should be actively working on practical strategies to solve the life problems.

Essential	Important	Don't know/Depends	Unimportant	Should not be included
◯	◯	◯	◯	◯

212. If the suicidal person wants to be left alone, and can assure the first aider of their safety, the first aider should agree.

Essential	Important	Don't know/Depends	Unimportant	Should not be included
◯	◯	◯	◯	◯

Do you have any comments on these statements? Is there anything you would like to add to this section? Please write your ideas in the box provided

Section 8. What the first aider should know

The statements in this section are about the knowledge the first aider should have to enable them to provide mental health first aid to the suicidal person.

Please rate how important (from *essential* to *should not be included*) you think it is that each statement be included in the final guidelines.

The first aider should be aware:

	Essential	Important	Don't know/Depends	Unimportant	Should not be included
213. Of how commonly suicide occurs.	◯	◯	◯	◯	◯
214. That there are many more suicide attempts than suicides.	◯	◯	◯	◯	◯
215. Of the risk factors for suicide.	◯	◯	◯	◯	◯
216. Of the link between suicide and mental illness.	◯	◯	◯	◯	◯
217. That talking about suicide will not 'put the idea' into someone's head.	◯	◯	◯	◯	◯
218. Of the reasons why people have thoughts about suicide.	◯	◯	◯	◯	◯
219. That most suicidal people do not want to die. They simply do not want to live with the pain.	◯	◯	◯	◯	◯
220. That suicidal people believe they have no choice but to die by suicide.	◯	◯	◯	◯	◯
221. That anyone could have thoughts of suicide.	◯	◯	◯	◯	◯
222. That suicidal thoughts are temporary.	◯	◯	◯	◯	◯
223. That suicidal behaviour is a cry for help.	◯	◯	◯	◯	◯
224. Of the reasons why people who are having suicidal thoughts don't ask for help.	◯	◯	◯	◯	◯
225. That people thinking about suicide are not likely to seek help, but do show warning signs to their family and friends.	◯	◯	◯	◯	◯
226. That suicide can be prevented.	◯	◯	◯	◯	◯
227. That openly talking about suicidal thoughts and feelings can save a life.	◯	◯	◯	◯	◯
228. That they should not underestimate their abilities to help a suicidal person, even to save a life.	◯	◯	◯	◯	◯
229. That unless someone tells you, the only way to know if a person is thinking of suicide is to ask.	◯	◯	◯	◯	◯
230. That use of alcohol or other drugs can increase the risk of a person acting on suicidal thoughts.	◯	◯	◯	◯	◯
231. That even though the first aider can offer support, they are not responsible for the actions or behaviour of someone else, and cannot control what they might decide to do.	◯	◯	◯	◯	◯
232. Of the local services that can assist in response to people at risk of suicide (i.e. hospitals, mental health clinics, mobile outreach crisis teams, suicide prevention helplines, local emergency services).	◯	◯	◯	◯	◯

Do you have any comments on these statements? Is there anything you would like to add to this section? Please write your ideas in the box provided

Section 9. Confidentiality

The statements in this section are about the confidentiality of the suicidal person's thoughts and behaviours.

Please rate how important (from *essential* to *should not be included*) you think it is that each statement be included in the final guidelines.

233. The first aider must never agree to keep the risk of suicide a secret.

Essential	Important	Don't know/Depends	Unimportant	Should not be included
○	○	○	○	○

234. The first aider must never agree to keep the suicidal person's suicidal plans a secret.

Essential	Important	Don't know/Depends	Unimportant	Should not be included
○	○	○	○	○

235. If the suicidal person doesn't want the first aider to tell anyone about their suicidal thoughts, the first aider should not agree and explain why, e.g. "I care about you too much to keep a secret like this. You need help and I am here to help you get it".

Essential	Important	Don't know/Depends	Unimportant	Should not be included
○	○	○	○	○

236. If the suicidal person asks the first aider to keep the discussion about suicide a secret, the first aider should keep the secret.

Essential	Important	Don't know/Depends	Unimportant	Should not be included
○	○	○	○	○

237. If the suicidal person asks the first aider to promise they will keep the discussion about suicide a secret, they should agree, but tell someone else anyway.

Essential	Important	Don't know/Depends	Unimportant	Should not be included
○	○	○	○	○

238. The first aider should not keep the person's suicidal thoughts a secret from potential helpers, but should discuss with the person whether other details should be confidential.

Essential	Important	Don't know/Depends	Unimportant	Should not be included
◯	◯	◯	◯	◯

239. The first aider should treat everything the suicidal person says in complete confidence.

Essential	Important	Don't know/Depends	Unimportant	Should not be included
◯	◯	◯	◯	◯

240. The first aider should treat the suicidal person with respect and involve them in decisions about who else knows about the suicidal crisis.

Essential	Important	Don't know/Depends	Unimportant	Should not be included
◯	◯	◯	◯	◯

241. If the suicidal person refuses to give permission to disclose information about their suicidal thoughts, the first aider may need to breach their confidentiality to ensure their safety.

Essential	Important	Don't know/Depends	Unimportant	Should not be included
◯	◯	◯	◯	◯

242. The first aider should keep in mind that it is much better to have the person angry at them for sharing their suicidal thoughts without their permission in order to obtain help than to lose the person to suicide.

Essential	Important	Don't know/Depends	Unimportant	Should not be included
◯	◯	◯	◯	◯

Do you have any comments on these statements? Is there anything you would like to add to this section? Please write your ideas in the box provided

Section 10. Adolescent specific

Because the support provided to young people might be quite different to the support provided to an adult, the statements in this section are to be rated for **providing mental health first aid to an adolescent** . The term **adolescent** refers to a young person aged between 12 and 18 years (nominally the high school years).

Please rate how important (from *essential* to *should not be included*) you think it is that each statement be included in the final guidelines.

Assessing seriousness of suicide risk

242. If the suicidal adolescent says that the situation is not serious or that they can handle it on their own, the first aider should respect this.

Essential	Important	Don't know/Depends	Unimportant	Should not be included
○	○	○	○	○

Initial assistance

243. The first aider should not leave an adolescent who is feeling suicidal on their own.

Essential	Important	Don't know/Depends	Unimportant	Should not be included
○	○	○	○	○

244. The first aider does not need to be with the suicidal adolescent all the time, but should check on them regularly.

Essential	Important	Don't know/Depends	Unimportant	Should not be included
○	○	○	○	○

245. If the adolescent is suicidal, the first aider should:

	Essential	Important	Don't know/Depends	Unimportant	Should not be included
Discuss with the suicidal person what actions they should take to get help.	○	○	○	○	○
Encourage the suicidal person to get appropriate professional help as soon as possible (i.e. See a mental health professional or someone at a mental health service).	○	○	○	○	○
Seek the permission of the suicidal person to contact their regular doctor or mental health professional about their concerns.	○	○	○	○	○
Call a mental health centre or crisis telephone line and ask for advice on the situation.	○	○	○	○	○
Call a doctor, psychiatrist or other professional right away for the suicidal person.	○	○	○	○	○
Get the suicidal person to phone an emergency number (i.e. Emergency services, a suicide helpline, emergency mental health services).	○	○	○	○	○
Phone an emergency number **without letting the suicidal person know**.	○	○	○	○	○
Take the suicidal person to a hospital emergency department.	○	○	○	○	○

246. If the suicidal adolescent can't commit to stay safe, the first aider should:

	Essential	Important	Don't know/Depends	Unimportant	Should not be included
Discuss with the suicidal person what actions they should take to get help.	○	○	○	○	○
Encourage the suicidal person to get appropriate professional help as soon as possible (i.e. See a mental health professional or someone at a mental health service).	○	○	○	○	○
Seek the permission of the suicidal person to contact their regular doctor or mental health professional about their concerns.	○	○	○	○	○
Call a mental health centre or crisis telephone line and ask for advice on the situation.	○	○	○	○	○
Call a doctor, psychiatrist or other professional right away for the suicidal person.	○	○	○	○	○
Get the suicidal person to phone an emergency number (i.e. Emergency services, a suicide helpline, emergency mental health services).	○	○	○	○	○
Phone an emergency number **without letting the suicidal person know**.	○	○	○	○	○
Take the suicidal person to a hospital emergency department.	○	○	○	○	○

Section 10. Adolescent specific (2)

247. If the suicidal adolescent has a specific plan, the first aider should:

	Essential	Important	Don't know/Depends	Unimportant	Should not be included
Discuss with the suicidal person what actions they should take to get help.	○	○	○	○	○
Encourage the suicidal person to get appropriate professional help as soon as possible (i.e. See a mental health professional or someone at a mental health service).	○	○	○	○	○
Seek the permission of the suicidal person to contact their regular doctor or mental health professional about their concerns.	○	○	○	○	○
Call a mental health centre or crisis telephone line and ask for advice on the situation.	○	○	○	○	○
Call a doctor, psychiatrist or other professional right away for the suicidal person.	○	○	○	○	○
Get the suicidal person to phone an emergency number (i.e. Emergency services, a suicide helpline, emergency mental health services).	○	○	○	○	○
Phone an emergency number **without letting the suicidal person know**.	○	○	○	○	○
Take the suicidal person to a hospital emergency department.	○	○	○	○	○

248. If the suicidal adolescent has the means to carry out their suicide plan, the first aider should:

	Essential	Important	Don't know/Depends	Unimportant	Should not be included
Discuss with the suicidal person what actions they should take to get help.	○	○	○	○	○
Encourage the suicidal person to get appropriate professional help as soon as possible (i.e. See a mental health professional or someone at a mental health service).	○	○	○	○	○
Seek the permission of the suicidal person to contact their regular doctor or mental health professional about their concerns.	○	○	○	○	○
Call a mental health centre or crisis telephone line and ask for advice on the situation.	○	○	○	○	○
Call a doctor, psychiatrist or other professional right away for the suicidal person.	○	○	○	○	○
Get the suicidal person to phone an emergency number (i.e. Emergency services, a suicide helpline, emergency mental health services).	○	○	○	○	○
Phone an emergency number **without letting the suicidal person know**.	○	○	○	○	○
Take the suicidal person to a hospital emergency department.	○	○	○	○	○

249. If the suicidal adolescent does not agree to give the first aider the things they intend using to kill themselves, the first aider should:

	Essential	Important	Don't know/Depends	Unimportant	Should not be included
Discuss with the suicidal person what actions they should take to get help.	○	○	○	○	○
Encourage the suicidal person to get appropriate professional help as soon as possible (i.e. See a mental health professional or someone at a mental health service).	○	○	○	○	○
Seek the permission of the suicidal person to contact their regular doctor or mental health professional about their concerns.	○	○	○	○	○
Call a mental health centre or crisis telephone line and ask for advice on the situation.	○	○	○	○	○
Call a doctor, psychiatrist or other professional right away for the suicidal person.	○	○	○	○	○
Get the suicidal person to phone an emergency number (i.e. Emergency services, a suicide helpline, emergency mental health services).	○	○	○	○	○
Phone an emergency number **without letting the suicidal person know**.	○	○	○	○	○
Take the suicidal person to a hospital emergency department.	○	○	○	○	○

249. If the suicidal adolescent has attempted suicide in the past, the first aider should:

	Essential	Important	Don't know/Depends	Unimportant	Should not be included
Discuss with the suicidal person what actions they should take to get help.	◯	◯	◯	◯	◯
Encourage the suicidal person to get appropriate professional help as soon as possible (i.e. See a mental health professional or someone at a mental health service).	◯	◯	◯	◯	◯
Seek the permission of the suicidal person to contact their regular doctor or mental health professional about their concerns.	◯	◯	◯	◯	◯
Call a mental health centre or crisis telephone line and ask for advice on the situation.	◯	◯	◯	◯	◯
Call a doctor, psychiatrist or other professional right away for the suicidal person.	◯	◯	◯	◯	◯
Get the suicidal person to phone an emergency number (i.e. Emergency services, a suicide helpline, emergency mental health services).	◯	◯	◯	◯	◯
Phone an emergency number **without letting the suicidal person know**.	◯	◯	◯	◯	◯
Take the suicidal person to a hospital emergency department.	◯	◯	◯	◯	◯

Section 10. Adolescent specific (3)

251. If the suicidal adolescent is known to have a diagnosis of a mental illness, the first aider should:

	Essential	Important	Don't know/Depends	Unimportant	Should not be included
Discuss with the suicidal person what actions they should take to get help.	◯	◯	◯	◯	◯
Encourage the suicidal person to get appropriate professional help as soon as possible (i.e. See a mental health professional or someone at a mental health service).	◯	◯	◯	◯	◯
Seek the permission of the suicidal person to contact their regular doctor or mental health professional about their concerns.	◯	◯	◯	◯	◯
Call a mental health centre or crisis telephone line and ask for advice on the situation.	◯	◯	◯	◯	◯
Call a doctor, psychiatrist or other professional right away for the suicidal person.	◯	◯	◯	◯	◯
Get the suicidal person to phone an emergency number (i.e. Emergency services, a suicide helpline, emergency mental health services).	◯	◯	◯	◯	◯
Phone an emergency number **without letting the suicidal person know**.	◯	◯	◯	◯	◯
Take the suicidal person to a hospital emergency department.	◯	◯	◯	◯	◯

252. If the suicidal adolescent is psychotic, the first aider should:

	Essential	Important	Don't know/Depends	Unimportant	Should not be included
Discuss with the suicidal person what actions they should take to get help.	◯	◯	◯	◯	◯
Encourage the suicidal person to get appropriate professional help as soon as possible (i.e. See a mental health professional or someone at a mental health service).	◯	◯	◯	◯	◯
Seek the permission of the suicidal person to contact their regular doctor or mental health professional about their concerns.	◯	◯	◯	◯	◯
Call a mental health centre or crisis telephone line and ask for advice on the situation.	◯	◯	◯	◯	◯
Call a doctor, psychiatrist or other professional right away for the suicidal person.	◯	◯	◯	◯	◯
Get the suicidal person to phone an emergency number (i.e. Emergency services, a suicide helpline, emergency mental health services).	◯	◯	◯	◯	◯
Phone an emergency number **without letting the suicidal person know**.	◯	◯	◯	◯	◯
Take the suicidal person to a hospital emergency department.	◯	◯	◯	◯	◯

253. If the suicidal adolescent refuses professional help, the first aider should:

	Essential	Important	Don't know/Depends	Unimportant	Should not be included
Discuss with the suicidal person what actions they should take to get help.	◯	◯	◯	◯	◯
Encourage the suicidal person to get appropriate professional help as soon as possible (i.e. See a mental health professional or someone at a mental health service).	◯	◯	◯	◯	◯
Seek the permission of the suicidal person to contact their regular doctor or mental health professional about their concerns.	◯	◯	◯	◯	◯
Call a mental health centre or crisis telephone line and ask for advice on the situation.	◯	◯	◯	◯	◯
Call a doctor, psychiatrist or other professional right away for the suicidal person.	◯	◯	◯	◯	◯
Get the suicidal person to phone an emergency number (i.e. Emergency services, a suicide helpline, emergency mental health services).	◯	◯	◯	◯	◯
Phone an emergency number **without letting the suicidal person know**.	◯	◯	◯	◯	◯
Take the suicidal person to a hospital emergency department.	◯	◯	◯	◯	◯

254. If the suicidal adolescent is reluctant to seek help, the first aider should:

	Essential	Important	Don't know/Depends	Unimportant	Should not be included
Keep encouraging them to see a mental health professional.	◯	◯	◯	◯	◯
Seek an agreement with them that they will contact a specific person within a specified timeframe.	◯	◯	◯	◯	◯
Contact a suicide prevention hotline for guidance on how to help them.	◯	◯	◯	◯	◯
Talk to a health professional for advice on the situation.	◯	◯	◯	◯	◯
Contact emergency services on their behalf.	◯	◯	◯	◯	◯
Make sure someone who is close to the suicidal adolescent is aware of the situation (i.e. close friend or family member).	◯	◯	◯	◯	◯

255. If the first aider is unable to persuade the suicidal adolescent to get help, they should get assistance from a trusted friend, helpline or mental health professional.

Essential	Important	Don't know/Depends	Unimportant	Should not be included
◯	◯	◯	◯	◯

256. The first aider should ensure that the suicidal adolescent receives help from a health professional, support group or relevant community organisation.

Essential	Important	Don't know/Depends	Unimportant	Should not be included
◯	◯	◯	◯	◯

Talking with a suicidal adolescent

257. The first aider should avoid giving advice to the suicidal adolescent.

Essential	Important	Don't know/Depends	Unimportant	Should not be included
◯	◯	◯	◯	◯

258. In order to reduce suicide risk, it is important for the first aider to try to solve the suicidal adolescent's problems.

Essential	Important	Don't know/Depends	Unimportant	Should not be included
◯	◯	◯	◯	◯

259. The first aider should not try to take on the suicidal adolescent's responsibilities.

Essential	Important	Don't know/Depends	Unimportant	Should not be included
○	○	○	○	○

No-suicide contracts

260. The first aider should develop a safety plan with the suicidal adolescent.

Essential	Important	Don't know/Depends	Unimportant	Should not be included
○	○	○	○	○

261. If the suicidal adolescent won't make a safety plan, it is not safe to leave them alone for any period of time. The first aider should make sure someone stays close by the person (in the same room, in visual contact) and get whatever outside resources are available (e.g. family, emergency mental health care or if necessary, the police).

Essential	Important	Don't know/Depends	Unimportant	Should not be included
○	○	○	○	○

262. If the suicidal adolescent won't make a safety plan, the first aider should get professional help immediately.

Essential	Important	Don't know/Depends	Unimportant	Should not be included
○	○	○	○	○

Passing time during the crisis

263. If the suicidal adolescent wants to be left alone, and can assure the first aider of their safety, the first aider should agree.

Essential	Important	Don't know/Depends	Unimportant	Should not be included
○	○	○	○	○

Confidentiality

264. The first aider should treat the suicidal adolescent with respect and involve them in decisions about who else knows about the suicidal crisis.

Essential	Important	Don't know/Depends	Unimportant	Should not be included
◯	◯	◯	◯	◯

265. If the suicidal adolescent asks the first aider to promise they will keep the discussion about suicide a secret, they should agree, but tell someone else anyway.

Essential	Important	Don't know/Depends	Unimportant	Should not be included
◯	◯	◯	◯	◯

Do you have any comments on these statements? Is there anything you would like to add to this section? Please write your ideas in the box provided

Round 1 survey complete!

That is the end of the first round survey! Thank you very much for your contribution.

By pressing the "done" button your final responses will be registered with our survey software. Once all panel members have lodged their responses, we will collate the data and send you a report on the findings.

Remember, if the statements presented have caused you to **feel sad or distressed in any way**, please do not hesitate to talk to someone about it. We recommend calling one of the helplines listed below:

Calling from Australia

beyond blue: 1300 22 4636 (24hrs)

LifeLine: 13 11 14 (24hrs, free call from mobile phone)

Calling from the USA

National Suicide Prevention Lifeline: 1800 273 8255 (24hrs, toll free)

National Hopeline Network: 1800 442 4673 (24hrs)

Calling from Canada

The support network: 780 482 4357 (24hrs)

Crisis Line: 1866 996 0991 (24hrs, toll free)

Salvation Army Suicide Prevention crisis line: 905 522 1477 (24hrs)

Calling from New Zealand

Lifeline: 0800 543 354 (24hrs, toll free)

Samaritans: 0800 726 666 (24hrs, toll free)

Calling from the UK

Samaritans: 08457 90 90 90 (24hrs)

SANEline: 0845 767 8000 (6pm-11pm)

Thank you once again. We are extremely grateful for your contribution.

The Research Team
