# Supplementary material for: Re-development of mental health first aid guidelines for suicidal ideation and behaviour: a Delphi study
Source: BMC Psychiatry. 2014 Sep 13;14:241. doi: 10.1186/s12888-014-0241-8 (PMC4199061; doi:10.1186/s12888-014-0241-8)
Supplement: Additional file 2: — Round 2 questionnaire. [file 12888_2014_241_MOESM2_ESM.pdf]

Redevelopment of mental health first aid guidelines for suicidal thoughts

Introduction

Purpose of the research

The aim of this project is to re-develop guidelines for members of the public providing first aid for people who are having suicidal thoughts or displaying suicidal behaviour. This redevelopment will ensure the guidelines remain consistent with the current literature and contain the most recent and appropriate helping actions, as well as continue to meet the standards for their inclusion on the NHMRC Clinical Practice Guidelines Portal.

As a first step, we are seeking to find current consensus on the key skills and knowledge that a person needs to help someone who is having suicidal thoughts or displaying suicidal behaviour. The statements from this questionnaire that receive a high level of consensus will be included in the mental health first aid guidelines for suicidal thoughts. The guidelines will then be used as the basis for an intervention training program.

Your role

You have been selected as a panel member for this study because you have expertise in the area of suicide prevention. Your task is to rate the statements presented in this questionnaire according to how important you believe they are to the aims of mental health first aid and the role of the first aider.

The role of a Mental Health First Aider is to help a person who is developing a mental health problem or is in a mental health crisis. The first aid is given until appropriate professional treatment is received or the crisis resolves.

How this questionnaire was developed

1. Items that appeared in Round 1 and are presented again in Round 2 for re-rating because they received a level of consensus that was neither high enough to be clearly endorsed nor low enough to be clearly rejected.

2. Items that appear for the first time in Round 2 which have been developed from comments made by panel members in Round 1(**denoted by an ***).

It is important to note that we do not necessarily agree with these statements; we have included them because we do not believe that we should decide what the best practice is for mental health first aid. Rather, we have invited you to be a member of the expert panel to help develop a set of guidelines that reflect current expert opinion across the field of suicide prevention.

Other instructions to note

This questionnaire should take approximately **15-20 minutes** to complete. However, some people may finish it much more quickly. Others may take more time. Please be aware that once you have logged on and started responding, you may save your answers at any time by completing a page and clicking "Next" at the bottom. This marks your page as complete and you may begin again another time at the next page. Please make sure that you always log back in using the same email link, otherwise the software will fail to recognise your code and previously saved

responses.

We would like to **thank you** for your time and effort and encourage you to provide us with feedback on this process.

Best wishes,

The Research Team
Population Mental Health Group
Melbourne School of Population and Global Health
University of Melbourne

Definitions of terms used in this questionnaire

Throughout the questionnaire, we use the terms **'the person'** to refer to the person experiencing suicidal thoughts or engaging in non-suicidal self-injury, and **'the first aider'** to refer to the person providing assistance.

Mental health problem is a broad term that includes developing mental illness, symptoms of a diagnosable illness, substance use, and adverse life events which are having an impact on functioning. A **mental illness** is a mental health problem that has been diagnosed by a mental health professional, that affects a person's thinking, emotional state and behaviour, and disrupts a person's ability to work or carry out other daily activities and engage in satisfying personal relationships.

Emergency services refers to the best services available at short notice for the situation in the area. **Emergency mental health services** refers to mental health services that are available at short notice in the area, and could include a crisis team or a suicide helpline.

A **Safety plan** is an agreement between the suicidal person and the first aider that involves actions to keep the suicidal person safe.

Please be aware that the statements in this survey only apply to first aid given by adults. However, there are some statements specifically about the support that an adult could provide to an adolescent. These items are included in cases where the support provided to an adolescent might be quite different to the support provided to an adult. The term **adolescent** refers to a young person aged between 12 and 18 years (nominally the high school years).

Overview of suicide questionnaire content

Please note that there will be an opportunity to add any **comments or suggestions** you might have at the **end of each section** of the survey.

Section 1- Identification of suicide risk

- *Things to consider before making an approach*
- *How to approach the person of concern*
- *Asking about suicidal thoughts*
- *What to do if the person of concern doesn't want to talk*
- *What to do if the first aider feels unsuccessful in their approach*

- *What to do if the person of concern is psychotic*
- *What to do if the person of concern is under the influence of drugs and alcohol*

Section 2- Assessing seriousness of the suicide risk

- *Assessing the urgency of the risk*
- *Finding out about the suicidal person's plan*
- *Asking about the factors that contribute to risk*
- *Asking about other factors that contribute to risk*

Section 3- Initial assistance

- *Providing initial assistance to the suicidal person*

Section 4- Talking with the suicidal person

- *What to do when talking with the suicidal person*
- *Things to avoid when talking with the suicidal person*

Section 5- No-suicide contracts

- *Developing a safety plan*
- *What the safety plan should include*
- *Timeframe of the safety plan*
- *When to make a safety plan*

Section 6- Ensuring safety

- *Reducing the chances of the suicidal person acting on suicidal thoughts*

Section 7- Passing time during the crisis

- *What to do until the suicide crisis passes*

Section 8- What the first aider should know

- *The facts about suicide*
- *The preventable nature of suicide*

Section 9- Confidentiality

- *Determining what information needs to be shared with others*

Section 10- Adolescent specific

- *Helping statements that may differ in endorsement for people aged 12-18 compared to adults*

Section 1. Identification of suicide risk

This section contains statements about identifying the severity of the person's risk of suicide.

NOTE: If the person has already acted on their thoughts of suicide, this should be treated as a medical emergency.

Please rate how important (from *essential* to *should not be included*) you think it is that each statement be included in the final guidelines.

Please note that there will be an opportunity to add any **comments or suggestions** you might have at the **end of each section** of the survey.

Before approaching the person

4. If the suicidal person has already harmed themselves, the first aider should administer first aid and call emergency services, asking for an ambulance.

Essential	Important	Don't know/Depends	Unimportant	Should not be included
○	○	○	○	○

8. The first aider should choose a private place to talk the person about their concerns.

Essential	Important	Don't know/Depends	Unimportant	Should not be included
○	○	○	○	○

How to make the approach

The first aider should:

	Essential	Important	Don't know/Depends	Unimportant	Should not be included
10. Tell the person their concerns about them, describing behaviours that have caused them to be concerned about suicide.	○	○	○	○	○
13. Demonstrate appropriate language when referring to suicide by using the terms 'suicide' or 'died by suicide'.	○	○	○	○	○

Asking about suicidal thoughts

18. The first aider should ask the person about suicidal thoughts, even if the first aider feels uncomfortable doing so.

Essential	Important	Don't know/Depends	Unimportant	Should not be included
○	○	○	○	○

***22.1. The first aider should not ask about suicide in leading or judgemental ways e.g. 'You're not thinking of doing anything stupid, are you?'**

Essential	Important	Don't know/Depends	Unimportant	Should not be included
○	○	○	○	○

The first aider should be aware that:

	Essential	Important	Don't know/Depends	Unimportant	Should not be included
23. If a person is not suicidal, asking them cannot put the idea of suicide in their head.	○	○	○	○	○

If the person doesn't want to talk

31. If the first aider thinks the person is uncomfortable interacting with them due to differences in age group or gender, they should ask the person if they would prefer to talk to someone of the same age group or gender.

Essential	Important	Don't know/Depends	Unimportant	Should not be included
◯	◯	◯	◯	◯

32. If the first aider thinks the person is uncomfortable interacting with them due to differences in cultural background, they should ask the person if they would prefer to talk to someone of the same cultural background.

Essential	Important	Don't know/Depends	Unimportant	Should not be included
◯	◯	◯	◯	◯

***32.1. The first aider should be aware that it is more important to genuinely want to help than to be of the same age, gender or cultural background as the person.**

Essential	Important	Don't know/Depends	Unimportant	Should not be included
◯	◯	◯	◯	◯

Section 1. Identification of suicide risk continued (2)

Reacting to expressions of suicidal thoughts

The first aider should:

	Essential	Important	Don't know/Depends	Unimportant	Should not be included
36. Know that it is common to feel panic or shock when someone discloses thoughts of suicide.	◯	◯	◯	◯	◯
40. Respect the suicidal person and not try to take charge of the situation.	◯	◯	◯	◯	◯

43. If the person is at a point of despair, the first aider needs to take control and be directive in ensuring their safety.

Essential	Important	Don't know/Depends	Unimportant	Should not be included
◯	◯	◯	◯	◯

If the person is experiencing an episode of psychosis

***44.1. The first aider should tell the person that suicidal thoughts are temporary and will reduce over time.**

Essential	Important	Don't know/Depends	Unimportant	Should not be included
○	○	○	○	○

***44.2.The first aider should tell the person that they won't feel this badly forever.**

Essential	Important	Don't know/Depends	Unimportant	Should not be included
○	○	○	○	○

***46.1. If the suicidal person says they are hearing voices, the first aider should ask what the voices are telling the person in case the voices are relevant to their current suicidal thoughts.**

Essential	Important	Don't know/Depends	Unimportant	Should not be included
○	○	○	○	○

Section 2. Assessing seriousness of the suicide risk

This section contains statements about accessing the seriousness of the person's risk of suicide.

Please rate how important (from *essential* to *should not be included*) you think it is that each statement be included in the final guidelines.

Assessing the urgency of suicide risk

49. The first aider should determine the urgency of taking action based on recognition of suicide warning signs.

Essential	Important	Don't know/Depends	Unimportant	Should not be included
○	○	○	○	○

52. The first aider should establish whether the person has definite plans and intentions to take their life as opposed to vague suicidal notions such as "what's the point?" or "I can't be bothered going on".

Essential	Important	Don't know/Depends	Unimportant	Should not be included
○	○	○	○	○

Finding out about a suicide plan

The first aider should:

	Essential	Important	Don't know/Depends	Unimportant	Should not be included
56. Ask the suicidal person how they intend to suicide i.e. ask them direct questions about how, when and where they intend to suicide.	○	○	○	○	○

Asking about other factors that contribute to risk

The first aider should ask the suicidal person:

	Essential	Important	Don't know/Depends	Unimportant	Should not be included
63. If they have received mental health treatment in the past.	○	○	○	○	○
*63.1. If they have ever received help from a mental health professional.	○	○	○	○	○
*63.2. If they have a serious physical illness or if they are experiencing chronic pain.	○	○	○	○	○
*64.1. If anyone who they felt close to has made a suicide attempt.	○	○	○	○	○
*64.2. If anyone who they felt close to has died by suicide.	○	○	○	○	○
66. If they have ever made a suicide plan in the past.	○	○	○	○	○
70. How things are at home and work/school.	○	○	○	○	○
71. If there have been changes in their employment, social life, or family.	○	○	○	○	○
*71.1. Whether the person has major debts (e.g. gambling losses).	○	○	○	○	○

***63.3. If the person has been helped by a mental health professional in the past, the first aider should encourage them to reconnect with that person.**

Essential	Important	Don't know/Depends	Unimportant	Should not be included
○	○	○	○	○

Section 3. Initial assistance

This section contains statements about the assistance to be first provided to the person.

Please rate how important (from *essential* to *should not be included*) you think it is that each statement be included in the final guidelines.

75. The first aider should not put themselves in any danger while offering support to the suicidal person.

Essential	Important	Don't know/Depends	Unimportant	Should not be included
○	○	○	○	○

76. The first aider should not leave someone who is feeling suicidal on their own.

Essential	Important	Don't know/Depends	Unimportant	Should not be included
○	○	○	○	○

81. If the person is suicidal, the first aider should:

	Essential	Important	Don't know/Depends	Unimportant	Should not be included
Discuss with the suicidal person what actions they should take to get help.	○	○	○	○	○

82. If the suicidal person can't commit to stay safe, the first aider should:

	Essential	Important	Don't know/Depends	Unimportant	Should not be included
Encourage the suicidal person to get appropriate professional help as soon as possible (i.e. See a mental health professional or someone at a mental health service).	○	○	○	○	○
Seek the permission of the suicidal person to contact their regular doctor or mental health professional about their concerns.	○	○	○	○	○
Call a mental health centre or crisis telephone line and ask for advice on the situation.	○	○	○	○	○

83. If the suicidal person has a specific plan, the first aider should:

	Essential	Important	Don't know/Depends	Unimportant	Should not be included
Call a mental health centre or crisis telephone line and ask for advice on the situation.	○	○	○	○	○

84. If the suicidal person has the means to carry out their suicide plan, the first aider should:

	Essential	Important	Don't know/Depends	Unimportant	Should not be included
Encourage the suicidal person to get appropriate professional help as soon as possible (i.e. See a mental health professional or someone at a mental health service).	○	○	○	○	○
Seek the permission of the suicidal person to contact their regular doctor or mental health professional about their concerns.	○	○	○	○	○
Call a mental health centre or crisis telephone line and ask for advice on the situation.	○	○	○	○	○

85. If the suicidal person does not agree to give the first aider the things they intend using to kill themselves, the first aider should:

	Essential	Important	Don't know/Depends	Unimportant	Should not be included
Discuss with the suicidal person what actions they should take to get help.	○	○	○	○	○
Encourage the suicidal person to get appropriate professional help as soon as possible (i.e. See a mental health professional or someone at a mental health service).	○	○	○	○	○
Seek the permission of the suicidal person to contact their regular doctor or mental health professional about their concerns.	○	○	○	○	○
Call a mental health centre or crisis telephone line and ask for advice on the situation.	○	○	○	○	○
Call a doctor, psychiatrist or other professional right away for the suicidal person.	○	○	○	○	○
Get the suicidal person to phone an emergency number (i.e. Emergency services, a suicide helpline, emergency mental health services).	○	○	○	○	○
Phone an emergency number **without letting the suicidal person know**.	○	○	○	○	○
Take the suicidal person to a hospital emergency department.	○	○	○	○	○

86. If the suicidal person has attempted suicide in the past, the first aider should:

	Essential	Important	Don't know/Depends	Unimportant	Should not be included
Call a mental health centre or crisis telephone line and ask for advice on the situation.	○	○	○	○	○

Section 3. Initial assistance (2)

87. If the suicidal person is known to have a diagnosis of a mental illness, the first aider should:

	Essential	Important	Don't know/Depends	Unimportant	Should not be included
Seek the permission of the suicidal person to contact their regular doctor or mental health professional about their concerns.	○	○	○	○	○

88. If the suicidal person is psychotic, the first aider should:

	Essential	Important	Don't know/Depends	Unimportant	Should not be included
Call a mental health centre or crisis telephone line and ask for advice on the situation.	○	○	○	○	○

89. If the suicidal person refuses professional help, the first aider should:

	Essential	Important	Don't know/Depends	Unimportant	Should not be included
Encourage the suicidal person to get appropriate professional help as soon as possible (i.e. See a mental health professional or someone at a mental health service).	○	○	○	○	○
Call a mental health centre or crisis telephone line and ask for advice on the situation.	○	○	○	○	○

92. If the suicidal person is reluctant to seek help, the first aider should:

	Essential	Important	Don't know/Depends	Unimportant	Should not be included
Talk to a health professional for advice on the situation.	○	○	○	○	○
Make sure someone who is close to the suicidal person is aware of the situation (i.e. close friend or family member).	○	○	○	○	○

93. If the suicidal person doesn't want to talk to someone face-to-face, the first aider should encourage them to contact a suicide helpline.

Essential	Important	Don't know/Depends	Unimportant	Should not be included
○	○	○	○	○

97. If the suicidal person has a weapon, the first aider should:

	Essential	Important	Don't know/Depends	Unimportant	Should not be included
Contact the police.	○	○	○	○	○

*98.1. The first aider should exercise appropriate self-care after helping someone who is feeling suicidal.

Essential	Important	Don't know/Depends	Unimportant	Should not be included
○	○	○	○	○

Section 4. Talking with a suicidal person

This section contains statements about talking to the suicidal person about their suicidal thoughts.

Please rate how important (from *essential* to *should not be included*) you think it is that each statement be included in the final guidelines.

Letting them know you care

The first aider should:

	Essential	Important	Don't know/Depends	Unimportant	Should not be included
109. Keep in mind that asking too many questions can provoke anxiety in the suicidal person.	○	○	○	○	○

Actively listening

The first aider should:

	Essential	Important	Don't know/Depends	Unimportant	Should not be included
119. Share their thoughts with the suicidal person without expressing judgement.	◯	◯	◯	◯	◯

Reacting to suicidal thoughts

The first aider should:

	Essential	Important	Don't know/Depends	Unimportant	Should not be included
123. Encourage the suicidal person to discuss their reasons for dying and their reasons for living, validate that they are considering both options and emphasise that living is an option for them.	◯	◯	◯	◯	◯

Offering reassurance

The first aider should:

	Essential	Important	Don't know/Depends	Unimportant	Should not be included
129. Reassure the suicidal person that it's okay to feel the way they do.	◯	◯	◯	◯	◯
131. Offer hope and reassurance that their feelings are temporary, that help is available and things will get better.	◯	◯	◯	◯	◯
137. Remind the suicidal person that suicidal thoughts need not be acted on.	◯	◯	◯	◯	◯

Highlighting protective factors

158. The first aider should not touch (e.g. hug or hold hands with) the suicidal person without their permission.

Essential	Important	Don't know/Depends	Unimportant	Should not be included
◯	◯	◯	◯	◯

What to avoid when talking with a suicidal person

164. The first aider should NOT:

	Essential	Important	Don't know/Depends	Unimportant	Should not be included
Interrupt with stories of their own.	◯	◯	◯	◯	◯

Section 5. No-suicide contracts & safety planning

The statements in this section are about the development of no-suicide contracts and planning to keep the suicidal

person safe.

Please keep in mind that a **Safety plan** is an agreement between the suicidal person and the first aider that involves actions to keep the suicidal person safe.

Please rate how important (from *essential* to *should not be included*) you think it is that each statement be included in the final guidelines.

Developing a safety plan

169. The first aider should engage the suicidal person to the fullest extent possible in decisions about a safety plan.

Essential	Important	Don't know/Depends	Unimportant	Should not be included
○	○	○	○	○

What the plan should include

The safety plan should:

	Essential	Important	Don't know/Depends	Unimportant	Should not be included
174. Be clear, outlining what will be done, who will be doing it, and when it will be carried out.	○	○	○	○	○
175. Focus more on what the suicidal person should do rather than what they should not do.	○	○	○	○	○

Timeframe of the safety plan

The safety plan should:

	Essential	Important	Don't know/Depends	Unimportant	Should not be included
184. Be for a length of time which will be easy for the suicidal person to cope with, so that they can feel able to fulfil the agreement and have a sense of achievement.	○	○	○	○	○

Section 6. Ensuring safety

The statements in this section contain actions the first aider can take to help ensure the safety of the suicidal person.

Please rate how important (from *essential* to *should not be included*) you think it is that each statement be included in the final guidelines.

195. The first aider should try to remove the means of suicide available to the suicidal person if it is safe to do so.

Essential	Important	Don't know/Depends	Unimportant	Should not be included
○	○	○	○	○

***195.1. The first aider should try to remove the person from the means of suicide e.g. if the suicidal person is at home alone with the means for suicide, the first aider should take them somewhere safe.**

Essential	Important	Don't know/Depends	Unimportant	Should not be included
○	○	○	○	○

Section 7. Passing time during a crisis

The statements in this section are about what the first aider should do with the suicidal person during the suicide crisis period.

Please rate how important (from *essential* to *should not be included*) you think it is that each statement be included in the final guidelines.

The first aider should:

	Essential	Important	Don't know/Depends	Unimportant	Should not be included
*202.1. Stay with the suicidal person and keep them company.	○	○	○	○	○
*202.2. Discuss with the person what might be helpful for them to pass time during the crisis.	○	○	○	○	○

Section 8. What the first aider should know

The statements in this section are about the knowledge the first aider should have to enable them to provide mental health first aid to the suicidal person.

Please rate how important (from *essential* to *should not be included*) you think it is that each statement be included in the final guidelines.

The first aider should be aware:

	Essential	Important	Don't know/Depends	Unimportant	Should not be included
213. Of how commonly suicide occurs.	○	○	○	○	○
214. That there are many more suicide attempts than suicides.	○	○	○	○	○
*214.1. That many people who are suicidal act impulsively and that the first aider's role is to slow down their thinking and help them feel more in control.	○	○	○	○	○
*217.1. That it is more important to be genuinely caring than to say 'all the right things'.	○	○	○	○	○
220. That suicidal people believe they have no choice but to die by suicide.	○	○	○	○	○
222. That suicidal thoughts are temporary.	○	○	○	○	○
229. That unless someone tells you, the only way to know if a person is thinking of suicide is to ask.	○	○	○	○	○

Section 9. Confidentiality

The statements in this section are about the confidentiality of the suicidal person's thoughts and behaviours.

Please rate how important (from *essential* to *should not be included*) you think it is that each statement be included in the final guidelines.

235. If the suicidal person doesn't want the first aider to tell anyone about their suicidal thoughts, the first aider should not agree and explain why, e.g. "I care about you too much to keep a secret like this. You need help and I am here to help you get it".

Essential	Important	Don't know/Depends	Unimportant	Should not be included
○	○	○	○	○

***235.1. If the first aider needs to breach confidentiality to ensure the person's safety, they should be transparent and tell the person who they will be notifying.**

Essential	Important	Don't know/Depends	Unimportant	Should not be included
○	○	○	○	○

***235.2. If the first aider needs to breach confidentiality to ensure the person's safety, they should go back to the person at a later time and tell them why they did it.**

Essential	Important	Don't know/Depends	Unimportant	Should not be included
○	○	○	○	○

Section 10. Adolescent specific

Because the support provided to young people might be quite different to the support provided to an adult, the statements in this section are to be rated for **providing mental health first aid to an adolescent** . The term **adolescent** refers to a young person aged between 12 and 18 years (nominally the high school years).

Please rate how important (from *essential* to *should not be included*) you think it is that each statement be included in the final guidelines.

Initial assistance

243. The first aider should not leave an adolescent who is feeling suicidal on their own.

Essential	Important	Don't know/Depends	Unimportant	Should not be included
○	○	○	○	○

***245. If the adolescent is suicidal, the first aider should:**

	Essential	Important	Don't know/Depends	Unimportant	Should not be included
Always notify the adolescent's parents that the adolescent is suicidal.	○	○	○	○	○

246. If the suicidal adolescent can't commit to stay safe, the first aider should:

	Essential	Important	Don't know/Depends	Unimportant	Should not be included
Encourage the suicidal person to get appropriate professional help as soon as possible (i.e. See a	○	○	○	○	○

Section 10. Adolescent specific (2)

247. If the suicidal adolescent has a specific plan, the first aider should:

	Essential	Important	Don't know/Depends	Unimportant	Should not be included
Call a mental health centre or crisis telephone line and ask for advice on the situation.	○	○	○	○	○

248. If the suicidal adolescent has the means to carry out their suicide plan, the first aider should:

	Essential	Important	Don't know/Depends	Unimportant	Should not be included
Discuss with the suicidal person what actions they should take to get help.	○	○	○	○	○
Encourage the suicidal person to get appropriate professional help as soon as possible (i.e. See a mental health professional or someone at a mental health service).	○	○	○	○	○
Call a mental health centre or crisis telephone line and ask for advice on the situation.	○	○	○	○	○
mber **without letting the suicidal person know**.	○	○	○	○	○
Take the suicidal person to a hospital emergency department.	○	○	○	○	○

249. If the suicidal adolescent does not agree to give the first aider the things they intend using to kill themselves, the first aider should:

	Essential	Important	Don't know/Depends	Unimportant	Should not be included
Discuss with the suicidal person what actions they should take to get help.	○	○	○	○	○
Encourage the suicidal person to get appropriate professional help as soon as possible (i.e. See a mental health professional or someone at a mental health service).	○	○	○	○	○

249. If the suicidal adolescent has attempted suicide in the past, the first aider should:

	Essential	Important	Don't know/Depends	Unimportant	Should not be included
Discuss with the suicidal person what actions they should take to get help.	○	○	○	○	○
Encourage the suicidal person to get appropriate professional help as soon as possible (i.e. See a mental health professional or someone at a mental health service).	○	○	○	○	○

251. If the suicidal adolescent is known to have a diagnosis of a mental illness, the first aider should:

	Essential	Important	Don't know/Depends	Unimportant	Should not be included
Seek the permission of the suicidal person to contact their regular doctor or mental health professional about their concerns.	○	○	○	○	○

253. If the suicidal adolescent refuses professional help, the first aider should:

	Essential	Important	Don't know/Depends	Unimportant	Should not be included
Call a mental health centre or crisis telephone line and ask for advice on the situation.	○	○	○	○	○

254. If the suicidal adolescent is reluctant to seek help, the first aider should:

	Essential	Important	Don't know/Depends	Unimportant	Should not be included
Keep encouraging them to see a mental health professional.	○	○	○	○	○
Contact a suicide prevention hotline for guidance on how to help them.	○	○	○	○	○

256. The first aider should ensure that the suicidal adolescent receives help from a health professional, support group or relevant community organisation.

Essential	Important	Don't know/Depends	Unimportant	Should not be included
○	○	○	○	○

259. The first aider should not try to take on the suicidal adolescent's responsibilities.

Essential	Important	Don't know/Depends	Unimportant	Should not be included
○	○	○	○	○

Round 2 survey complete!

That is the end of the second round survey! Thank you very much for your contribution.

By pressing the **"done" button** your final responses will be registered with our survey software. Once all panel members have lodged their responses, we will collate the data and send you a report on the findings.

Remember, if the statements presented have caused you to **feel sad or distressed in any way**, please do not hesitate to talk to someone about it. We recommend calling one of the helplines listed below:

Calling from Australia

beyond blue: 1300 22 4636 (24hrs)

LifeLine: 13 11 14 (24hrs, free call from mobile phone)

Calling from the USA

National Suicide Prevention Lifeline: 1800 273 8255 (24hrs, toll free)

National Hopeline Network: 1800 442 4673 (24hrs)

Calling from Canada

The support network: 780 482 4357 (24hrs)

Crisis Line: 1866 996 0991 (24hrs, toll free)

Salvation Army Suicide Prevention crisis line: 905 522 1477 (24hrs)

Calling from New Zealand

Lifeline: 0800 543 354 (24hrs, toll free)

Samaritans: 0800 726 666 (24hrs, toll free)

Calling from the UK

Samaritans: 08457 90 90 90 (24hrs)

SANEline: 0845 767 8000 (6pm-11pm)

Thank you once again. We are extremely grateful for your contribution.

The Research Team
