# Supplementary material for: Re-development of mental health first aid guidelines for suicidal ideation and behaviour: a Delphi study
Source: BMC Psychiatry. 2014 Sep 13;14:241. doi: 10.1186/s12888-014-0241-8 (PMC4199061; doi:10.1186/s12888-014-0241-8)
Supplement: Additional file 3: — Round 3 questionnaire. [file 12888_2014_241_MOESM3_ESM.pdf]

# Redevelopment of mental health first aid guidelines for suicidal thoughts

## Introduction

### Purpose of the research

The aim of this project is to re-develop guidelines for members of the public providing first aid for people who are having suicidal thoughts or displaying suicidal behaviour. This redevelopment will ensure the guidelines remain consistent with the current literature and contain the most recent and appropriate helping actions, as well as continue to meet the standards for their inclusion on the NHMRC Clinical Practice Guidelines Portal.

As a first step, we are seeking to find current consensus on the key skills and knowledge that a person needs to help someone who is having suicidal thoughts or displaying suicidal behaviour. The statements from this questionnaire that receive a high level of consensus will be included in the mental health first aid guidelines for suicidal thoughts. The guidelines will then be used as the basis for an intervention training program.

### Your role

You have been selected as a panel member for this study because you have expertise in the area of suicide prevention. Your task is to rate the statements presented in this questionnaire according to how important you believe they are to the aims of mental health first aid and the role of the first aider.

**The role of a Mental Health First Aider is to help a person who is developing a mental health problem or is in a mental health crisis. The first aid is given until appropriate professional treatment is received or the crisis resolves.**

### How this questionnaire was developed

The following questionnaire was derived from items that appeared in Round 2 for the first time (which were developed from comments made by panel members in Round 1) and are presented again in Round 3 for re-rating because they received a level of consensus that was neither high enough to be clearly endorsed nor low enough to be clearly rejected.

It is important to note that we do not necessarily agree with these statements; we have included them because we do not believe that we should decide what the best practice is for mental health first aid. Rather, we have invited you to be a member of the expert panel to help develop a set of guidelines that reflect current expert opinion across the field of suicide prevention.

### Other instructions to note

This questionnaire should take approximately **5 minutes** to complete, consisting of **only 3 items**. However, some people may finish it much more quickly. Others may take more time. Please be aware that once you have logged on and started responding, you may save your answers at any time by completing a page and clicking "Next" at the bottom. This marks your page as complete and you may begin again another time at the next page. Please make sure that you always log back in using the same email link, otherwise the software will fail to recognise your code and previously saved responses.

# Redevelopment of mental health first aid guidelines for suicidal thoughts

We would like to **thank you** for your time and effort and encourage you to provide us with feedback on this process.

Best wishes,

The Research Team
Population Mental Health Group
Melbourne School of Population and Global Health
University of Melbourne

## Definitions of terms used in this questionnaire

Throughout the questionnaire, we use the terms **'the person'** to refer to the person experiencing suicidal thoughts or engaging in non-suicidal self-injury, and **'the first aider'** to refer to the person providing assistance.

**Mental health problem** is a broad term that includes developing mental illness, symptoms of a diagnosable illness, substance use, and adverse life events which are having an impact on functioning. A **mental illness** is a mental health problem that has been diagnosed by a mental health professional, that affects a person's thinking, emotional state and behaviour, and disrupts a person's ability to work or carry out other daily activities and engage in satisfying personal relationships.

**Emergency services** refers to the best services available at short notice for the situation in the area. **Emergency mental health services** refers to mental health services that are available at short notice in the area, and could include a crisis team or a suicide helpline.

A **Safety plan** is an agreement between the suicidal person and the first aider that involves actions to keep the suicidal person safe.

Please be aware that the statements in this survey only apply to first aid given by adults. However, there are some statements specifically about the support that an adult could provide to an adolescent. These items are included in cases where the support provided to an adolescent might be quite different to the support provided to an adult. The term **adolescent** refers to a young person aged between 12 and 18 years (nominally the high school years).

## Overview of suicide questionnaire content

**Section 2- Assessing seriousness of the suicide risk**
• *Asking about other factors that contribute to risk*

**Section 7- Passing time during the crisis**
• *What to do until the suicide crisis passes*

## Section 2. Assessing seriousness of the suicide risk

This section contains statements about accessing the seriousness of the person's risk of suicide.

Please rate how important (from *essential* to *should not be included*) you think it is that each statement be included in the final guidelines.

***Asking about other factors that contribute to risk***

**The first aider should ask the suicidal person:**

| | Essential | Important | Don't know/Depends | Unimportant | Should not be included |
|---|---|---|---|---|---|
| 63.1 If they have ever received help from a mental health professional. | ○ | ○ | ○ | ○ | ○ |

## Section 7. Passing time during a crisis

The statements in this section are about what the first aider should do with the suicidal person during the suicide crisis period.

Please rate how important (from *essential* to *should not be included*) you think it is that each statement be included in the final guidelines.

**The first aider should:**

| | Essential | Important | Don't know/Depends | Unimportant | Should not be included |
|---|---|---|---|---|---|
| *202.1. Stay with the suicidal person and keep them company. | ○ | ○ | ○ | ○ | ○ |
| *202.2. Discuss with the person what might be helpful for them to pass time during the crisis. | ○ | ○ | ○ | ○ | ○ |

## Round 3 survey complete!

That is the end of the third and final survey! Thank you very much for your contribution across all three rounds. We are sincerely grateful to have your input into the development of such an important resource.

# Redevelopment of mental health first aid guidelines for suicidal thoughts

By pressing the **"done" button** your final responses will be registered with our survey software. Once all panel members have lodged their responses, we will collate the data and send you a report on the findings.

Remember, if the statements presented have caused you to **feel sad or distressed in any way**, please do not hesitate to talk to someone about it. We recommend calling one of the helplines listed below:

**Calling from Australia**

beyond blue: 1300 22 4636 (24hrs)

LifeLine: 13 11 14 (24hrs, free call from mobile phone)

**Calling from the USA**

National Suicide Prevention Lifeline: 1800 273 8255 (24hrs, toll free)

National Hopeline Network: 1800 442 4673 (24hrs)

**Calling from Canada**

The support network: 780 482 4357 (24hrs)

Crisis Line: 1866 996 0991 (24hrs, toll free)

Salvation Army Suicide Prevention crisis line: 905 522 1477 (24hrs)

**Calling from New Zealand**

Lifeline: 0800 543 354 (24hrs, toll free)

Samaritans: 0800 726 666 (24hrs, toll free)

**Calling from the UK**

Samaritans: 08457 90 90 90 (24hrs)

SANEline: 0845 767 8000 (6pm-11pm)

Thank you once again. We are extremely grateful for your contribution.

The Research Team
